# Identification of the N-terminal residues responsible for the differential microdomain localization of CYP1A1 and CYP1A2

**DOI:** 10.1016/j.jbc.2024.107891

**Published:** 2024-10-22

**Authors:** Robert M. Fuchs, James R. Reed, J. Patrick Connick, Markéta Paloncýová, Martin Šrejber, Petra Čechová, Michal Otyepka, Marilyn K. Eyer, Wayne L. Backes

**Affiliations:** 1 Department of Pharmacology and Experimental Therapeutics, and the Stanley S. Scott Cancer Center, Louisiana State University Health Sciences Center – New Orleans, New Orleans, Louisiana, USA; 2 Regional Center of Advanced Technologies and Materials, The Czech Advanced Technology and Research Institute (CATRIN), Palacký University Olomouc, Olomouc, Czech Republic; 3 IT4Innovations, VŠB – Technical University of Ostrava, Ostrava, Czech Republic

**Keywords:** cytochrome P450, protein-lipid interaction, membrane protein, structure-function, CYP1A1, CYP1A2, microdomain localization, microdomain-targeting motif, membrane charge depth, protein chimera

## Abstract

The endoplasmic reticulum is organized into ordered regions enriched in cholesterol and sphingomyelin, and disordered microdomains characterized by more fluidity. Rabbit CYP1A1 and CYP1A2 localize into disordered and ordered microdomains, respectively. Previously, a CYP1A2 chimera containing the first 109 amino acids of CYP1A1 showed altered microdomain localization. The goal of this study was to identify specific residues responsible for CYP1A microdomain localization. Thus, CYP1A2 chimeras containing substitutions from homologous regions of CYP1A1 were expressed in HEK 293T/17 cells, and the localization was examined after solubilization with Brij 98. A CYP1A2 mutant with the three amino acids from CYP1A1 (VAG) at positions 27 to 29 of CYP1A2 was generated that showed a distribution pattern similar to those of CYP1A1/1A2 chimeras containing both the first 109 amino acids and the first 31 amino acids of CYP1A1 followed by remaining amino acids of CYP1A2. Similarly, the reciprocal substitution of three amino acids from CYP1A2 (AVR) into CYP1A1 resulted in a partial redistribution of the chimera into ordered microdomains. Molecular dynamic simulations indicate that the positive charges of the CYP1A1 and CYP1A2 linker regions between the N termini and catalytic domains resulted in different depths of immersion of the N termini in the membrane. The overlap of the distribution of positively charged residues in CYP1A2 (AVR) and negatively charged phospholipids was higher in the ordered than in the disordered microdomain. These findings identify three residues in the CYP1AN terminus as a novel microdomain-targeting motif of the P450s and provide a mechanistic explanation for the differential microdomain localization of CYP1A.

The cytochromes P450 are a superfamily of heme-containing monooxygenases expressed in the liver, lung, intestine, and numerous other organs (1). These enzymes metabolize an enormous range of endogenous and xenobiotic substrates including hormones, hydrocarbons, and drugs (2). P450s can be targeted to multiple membranous organelles including the endoplasmic reticulum (ER), Golgi apparatus, and mitochondria (3, 4). Hepatic P450s involved in xenobiotic metabolism are bound to the ER membrane by the N terminus and an internal region near the F and G helices (5, 6). The membrane topology of microsomal P450s involves protrusion of the N terminus into the ER lumen, and in certain cases, the N-terminal amino acid sequence facilitates incorporation of the protein into the membrane (7, 8, 9, 10). However, studies have shown that some P450s truncated at the N terminus still attach to membranes, demonstrating that internal regions of the proteins also participate in their association with lipids (11, 12, 13). One possible identity for this internal region may be the F/G loop, which is present far downstream of the N terminus and embedded within the lipid bilayer (14).

Like other biologic membranes, the ER is composed of a heterogeneous lipid bilayer in which the lipids of each leaflet are segregated into regions with tight “ordered” packing in a surrounding disordered lipid milieu (15). Ordered lipid regions contain elevated levels of cholesterol and sphingomyelin, whereas the disordered regions are enriched in anionic phospholipids and unsaturated fatty acids (16). Specifically in the ER, disordered regions possess higher levels of phosphatidylcholine, phosphatidylethanolamine, and phosphatidylinositol, whereas ordered regions are relatively enriched in phosphatidylserine, cholesterol, and sphingomyelin (17, 18). In addition to their unique lipid compositions, ordered and disordered microdomains are enriched in different protein species (19). Experimentally, ordered and disordered membrane regions can be separated by selective detergent-based solubilization of disordered microdomains followed by ultracentrifugation to isolate the ordered membranes (6, 16, 20). These methods permit characterization of how membrane-bound proteins partition into ordered or disordered regions in biological membranes.

Different P450 enzymes were shown to reside in various ER microdomains (17, 21, 22, 23). Specifically, rabbit CYP1A2 is predominantly found in ordered microdomains, whereas CYP1A1 and CYP2E1 are in disordered regions—CYP2B4 distributes into both. Cholesterol depletion resulted in mobilization of CYP1A2 from predominantly ordered to disordered lipid microdomains, demonstrating that this protein preferentially resides in the ordered microdomains and that removal of the cholesterol framework allows this P450 to be solubilized by the nonionic detergent, Brij 98 (21, 24). Moreover, CYP1A2 also resides in detergent-resistant domains when incorporated into a purified, reconstituted system with the lipid composition of the total ER, providing even further evidence that it associates with ordered microdomains (24). The contrasting localization patterns of CYP1A1 and CYP1A2 were especially striking in light of their high sequence homology. To identify the general regions responsible for the different mobilization patterns of these proteins, chimeric CYP1A2/CYP1A1 fusion proteins were generated, and the microdomain localization patterns of these constructs were characterized (6). A chimeric CYP1A2 protein (containing only the first 109 amino acids of CYP1A1) strongly partitioned into the disordered region, similar to the pattern observed for WT CYP1A1. Conversely, chimeric CYP1A1 (containing the N terminal 107 residues of CYP1A2) showed a preference for ordered regions, similar to that of WT CYP1A2. Furthermore, chimeric CYP1A2, containing an internal region from CYP1A1 (amino acids 206–302), preferentially resided in the disordered region. These findings demonstrate that microdomain localization of the CYP1A proteins are governed by N-terminal residues and an internal region encompassing the F-G loop (6).

Due to the importance of the N-terminal region on the membrane localization of the CYP1A proteins, the goal of this study was to identify specific N-terminal residues responsible for the ordered lipid microdomain localization of CYP1A2. By aligning the sequences of CYP1A1 and CYP1A2, we found that most of the sequence differences in the first 110 amino acids were found within the first 30 positions of each protein. Therefore, CYP1A1 and CYP1A2 were aligned and a series of CYP1A2 chimeric constructs were generated where segments of the first 30 amino acids of CYP1A1 were substituted into the homologous region of CYP1A2. The sizes and regions of the substitutions were varied to identify the specific residues that caused relocalization of the mutated CYP1A2 into the disordered region. These studies identified a three amino-acid motif in the N terminus sufficient to influence CYP1A localization. Molecular dynamic (MD) simulations indicated that the positively charged amino acids in the linker regions connecting the N termini and the catalytic domains of CYP1A1 and CYP1A2 exist at different membrane depths when simulated in ordered and disordered regions. These differences in charge localization influence may explain the different microdomain localizations observed.

## Results

### Characterization of CYP1A transfection of HEK 293T/17 cells and Brij 98-mediated solubilization of PNS

**Figure 1:**
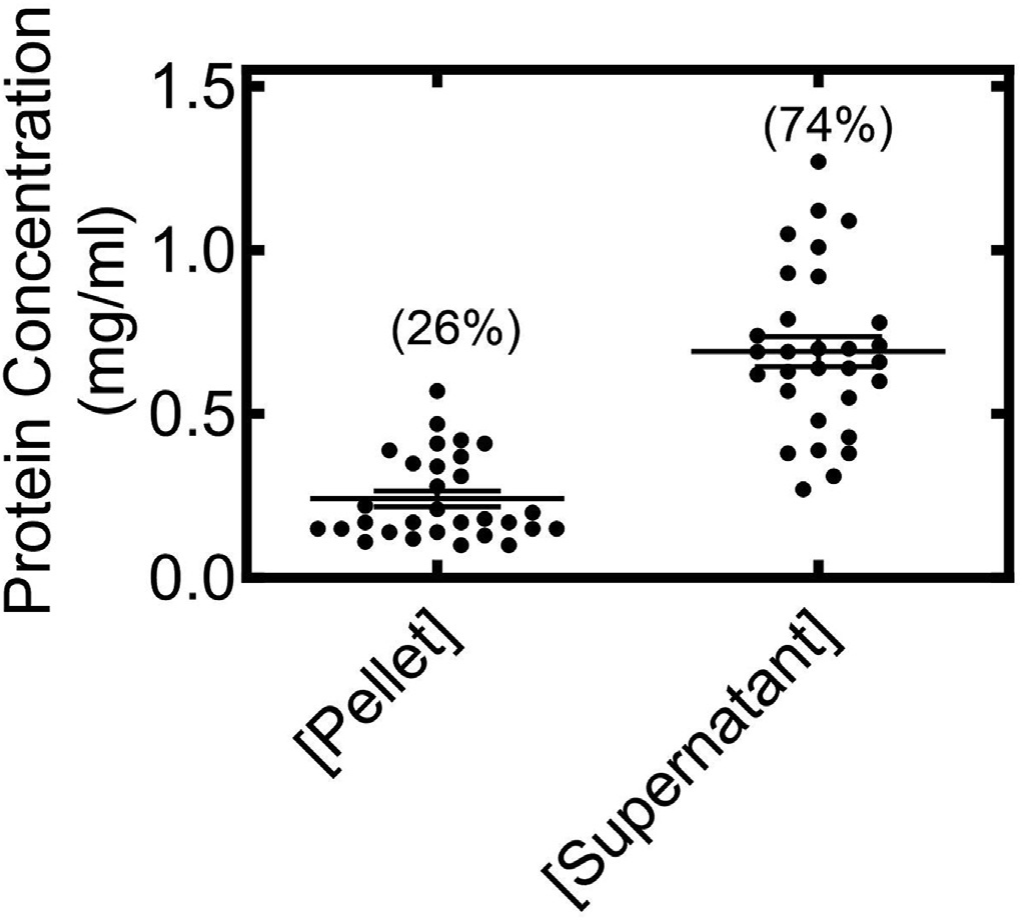
**Effect of Brij 98 solubilization on total microdomain distribution of PNS proteins derived from HEK 293T/17 cells.** CYP1A cDNA was transfected into HEK cells. The level of expression of CYP1A proteins was determined by PAGE. PNS samples from HEK cells were solubilized with 0.5% Brij 98, and detergent-soluble and detergent-resistant proteins were separated by ultracentrifugation as described in the Experimental procedures section. Protein concentrations of the different samples were determined by the BCA method. BCA, bicinchoninic acid; cDNA, complementary DNA; HEK, human embryonic kidney; PNS, postnuclear supernatant.

As preliminary experiments, we first examined the relative expression of CYP1 levels that were observed by transfection and to establish how transfection of CYPs would affect endogenous NADPH cytochrome P450 reductase (POR) expression (Fig. S1*A*). Although there was some variability in CYP1A expression, the levels of endogenous POR were about ten-fold lower than the transfected P450s and relatively resistant to CYP1 transfection. Additionally, changes in the expression levels of either P450 affected the endogenous expression of POR (Fig. S1*B*). When examining the distribution of proteins between the ordered (pellet) and disordered (supernatant) regions, the postnuclear supernatant (PNS) was roughly 1.5 mg/ml after three-fold dilution prior to ultracentrifugation (see Experimental procedures). Treatment with 0.5% Brij 98 (6, 21) led to the solubilization of 75% of the proteins, with 25% remaining in the pellet (Fig. 1).

### Detergent solubilization of chimeric and WT CYP1A forms

In previous reports, CYP1A1 was shown to preferentially reside in disordered membrane microdomains, whereas CYP1A2 localized in the ordered regions (6, 21). This was observed in both rabbit liver microsomes and in human embryonic kidney 293T/17 (HEK 293T/17) cells with transfected P450s. The differences in localization of these closely related proteins were shown to be due to differences in amino acid composition in two regions of the CYP1A proteins, the N terminus and the F-G loop. Although most of the residues in the first 100 amino acids of the N terminus were identical for CYP1A1 and CYP1A2, several differences were observed in the first 31 residues. In fact, generation of a GFP fusion protein that contained only the first 30 N-terminal residues from CYP1A2 caused it to localize in the ordered membrane region (6). These results point to the importance of the N terminus not only for membrane binding, but also on its microdomain localization.

**Figure 2:**
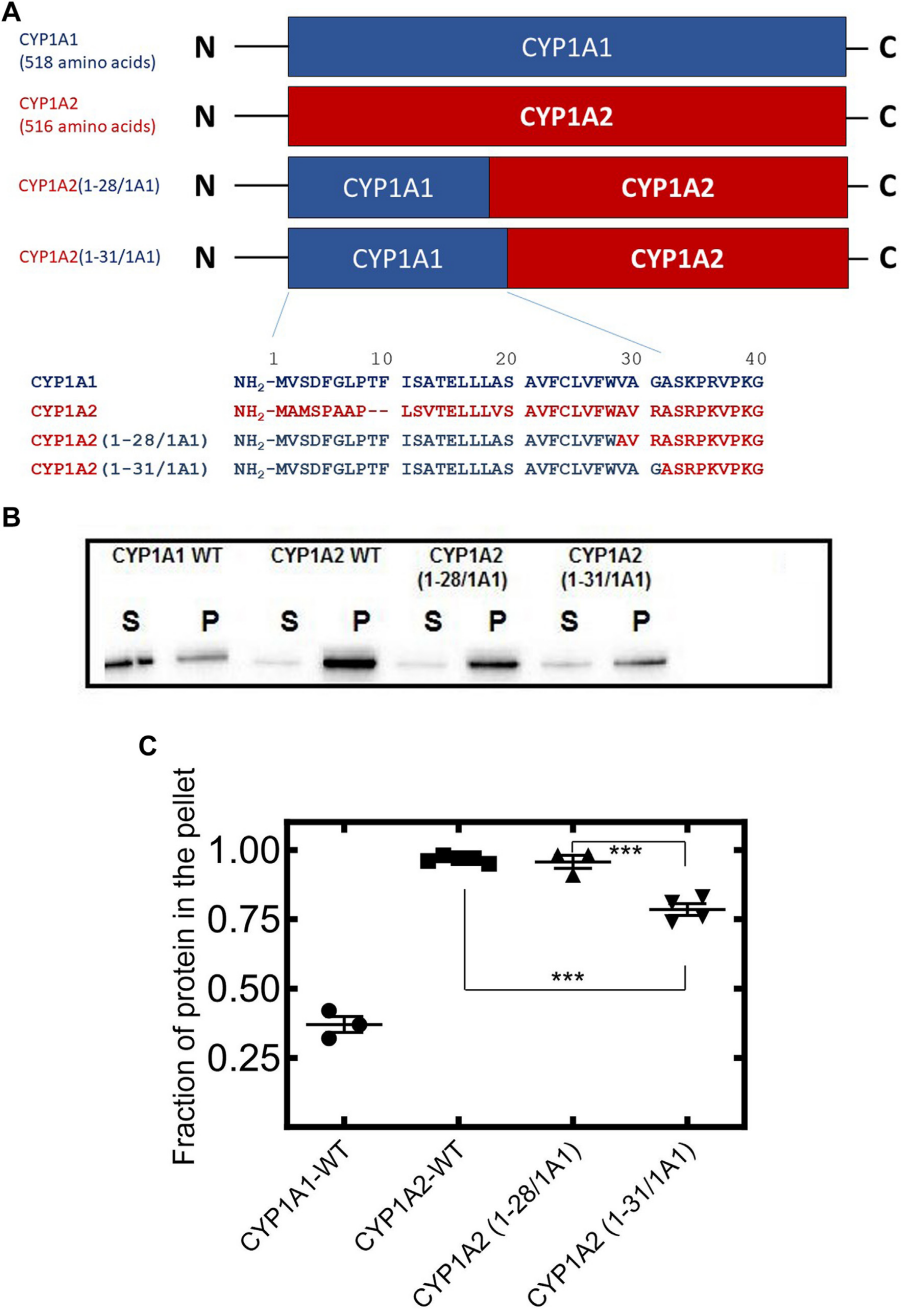
**Effect of N-terminal mutations of CYP1A2 on its microdomain localization.** WT CYP1A1 and CYP1A2 containing C-terminal GFP-tags and the chimeras, CYP1A2(1-28/1A1) and CYP1A2(1-31/1A1) were transfected into HEK 293T cells, and membrane localization was determined using Brij 98-based solubilization. *A*, comparison of amino acid sequences of WT CYP1A proteins, CYP1A2(1-28/1A1), and CYP1A2(1-31/1A1). *B*, distribution of WT and chimeric CYP1A proteins into detergent-soluble supernatants and detergent-resistant pellets following solubilization of HEK PNS with Brij 98 and ultracentrifugation (see Experimental procedures). The Brij solubilization images are a representative result in one of three different transfection experiments involving the PNS derived from 4 × 100 mm plates of HEK cells transfected with the indicated P450. *C*, quantitative results showing the percentages of proteins that were isolated in the detergent-resistant pellets following Brij 98-mediated solubilization of HEK PNS and ultracentrifugation. Each data point is the result from a single transfection experiment with each CYP1A form using four identical 100 mm plates of confluent HEK cells to generate the HEK PNS. Statistics were performed using a one-way analysis of variance and Bonferroni’s multiple comparison test (∗∗∗*p* < 0.001). HEK, human embryonic kidney; PNS, postnuclear supernatant.

**Figure 3:**
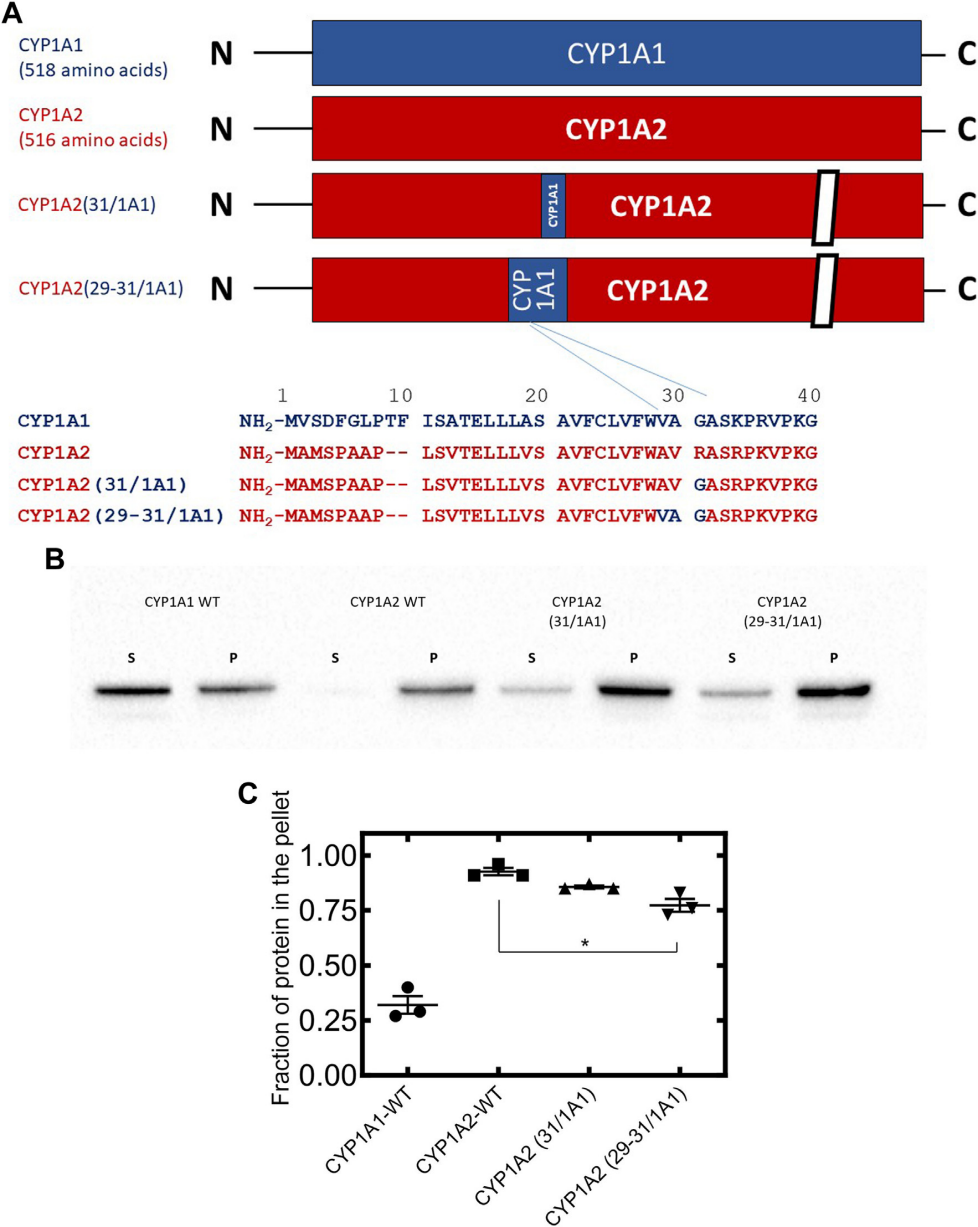
**Effect of N-terminal point mutations of CYP1A2 on its microdomain localization—WTCYP1A forms and the CYP1A2(31/1A1), and CYP1A2(29-31/1A1) chimeras.** Membrane localization of WT and chimeric CYP1A forms with C-terminal GFP-tags was determined after transfection of cDNA in HEK cells by Brij 98-based solubilization of PNS (see Experimental procedures section). Other than showing the results using different chimeric CYP1A forms shown, the legend to the figure is as described for Figure 2. *A*, comparison of amino acid sequences of WT CYP1A proteins, CYP1A2(31/1A1), and CYP1A2(29-31/1A1). *B*, distribution of WT and chimeric CYP1A proteins into detergent-soluble supernatants and detergent-resistant pellets following solubilization of HEK PNS with Brij 98 and ultracentrifugation (see Experimental procedures). The Brij solubilization images are a representative result in one of three different transfection experiments involving the PNS derived from 4 × 100 mm plates of HEK cells transfected with the indicated P450. *C*, quantitative results showing the percentages of proteins that was isolated in the detergent-resistant pellets following Brij 98-mediated solubilization of HEK PNS and ultracentrifugation. cDNA, complementary DNA; HEK, human embryonic kidney; PNS, postnuclear supernatant. Statistics were performed using a one-way ANOVA and Bonferroni’s multiple comparison test (∗*p* < 0.05).

The goal of this study was to identify, more specifically, the N-terminal residues that were responsible for the differential microdomain localization of CYP1A1 and CYP1A2. As a first step, chimeric proteins were generated that were primarily CYP1A2, but contained different sized substitutions of the N-terminal region from CYP1A1 (diagrammed in panel A from Figs. 2 and 3). CYP1A1, CYP1A2, and the chimeric proteins were transfected into HEK 293T cells, and the ordered and disordered regions were identified after Brij 98 treatment. The ordered lipid microdomains were isolated in the pellet upon ultracentrifugation, and proteins in the disordered regions were detected in the supernatant. As previously reported (6), Brij 98-treated CYP1A1 localized mainly in the disordered regions with CYP1A2 residing predominantly in the ordered microdomains, and substitution of the N-terminal 109 residues of CYP1A1 into CYP1A2 caused the chimeric protein to relocalize into the disordered domains. Replacement of the shorter 31 NH_2_-terminal amino acids of CYP1A1 into CYP1A2 also changed localization of the chimera more to the disordered microdomains (Fig. 2). However, when the smaller 28 amino acid NH_2_-terminal segment of CYP1A1 was substituted into CYP1A2, there was no detectable change in microdomain localization.

Other NH_2_-terminal substitutions were also examined. A CYP1A2 chimera that only contained residues 1–5 of CYP1A1 (Fig. S2) and a chimera of Ala for Val into position 13 did not affect microdomain localization.

### Identification of a disordered region-targeting motif in the first 31 amino acids of CYP1A1

When comparing the sequences between amino acid residues 5–31, there was a high level of agreement in the sequences of the two P450s (either identical or conserved amino acids); however, residues 27–29 of CYP1A2 (corresponding to residues 29–31 of CYP1A1) differed. In addition, these amino acids precede the polar, linker region of P450s, which is known to play an important role in orienting the proteins with respect to the negatively charged phosphate groups of lipids comprising the membrane. Therefore, we focused on this region as having the potential to modulate the microdomain localization of these CYP1A proteins. A chimera of CYP1A2 was generated that contained amino acids 29–31 of CYP1A1 in place of residues 27–29 of WT CYP1A2 (Fig. 3). This substitution caused the chimeric CYP1A2 to partially localize into the disordered microdomains in a manner that was similar to that observed for the CYP1A2 (1-31/1A1) chimera (Fig. 2). As the VAG tripeptide from CYP1A1 appeared to lead to different microdomain localization of the chimeric CYP1A2, the potential for the single nonconservative R to G amino acid substitution was examined. Although there appeared to be a minor effect on relocalization of CYP1A2 after substituting this single amino acid, the results were not significantly different from the localization of WT CYP1A2. Taken together, the results are consistent with residues 27–29 being involved in the microdomain localization of CYP1A2, but not solely the nonconservative R→G substitution.

### Reciprocal experiment—effect of substitution of residues 27–29 from CYP1A2 into CYP1A1 on microdomain localization

**Figure 4:**
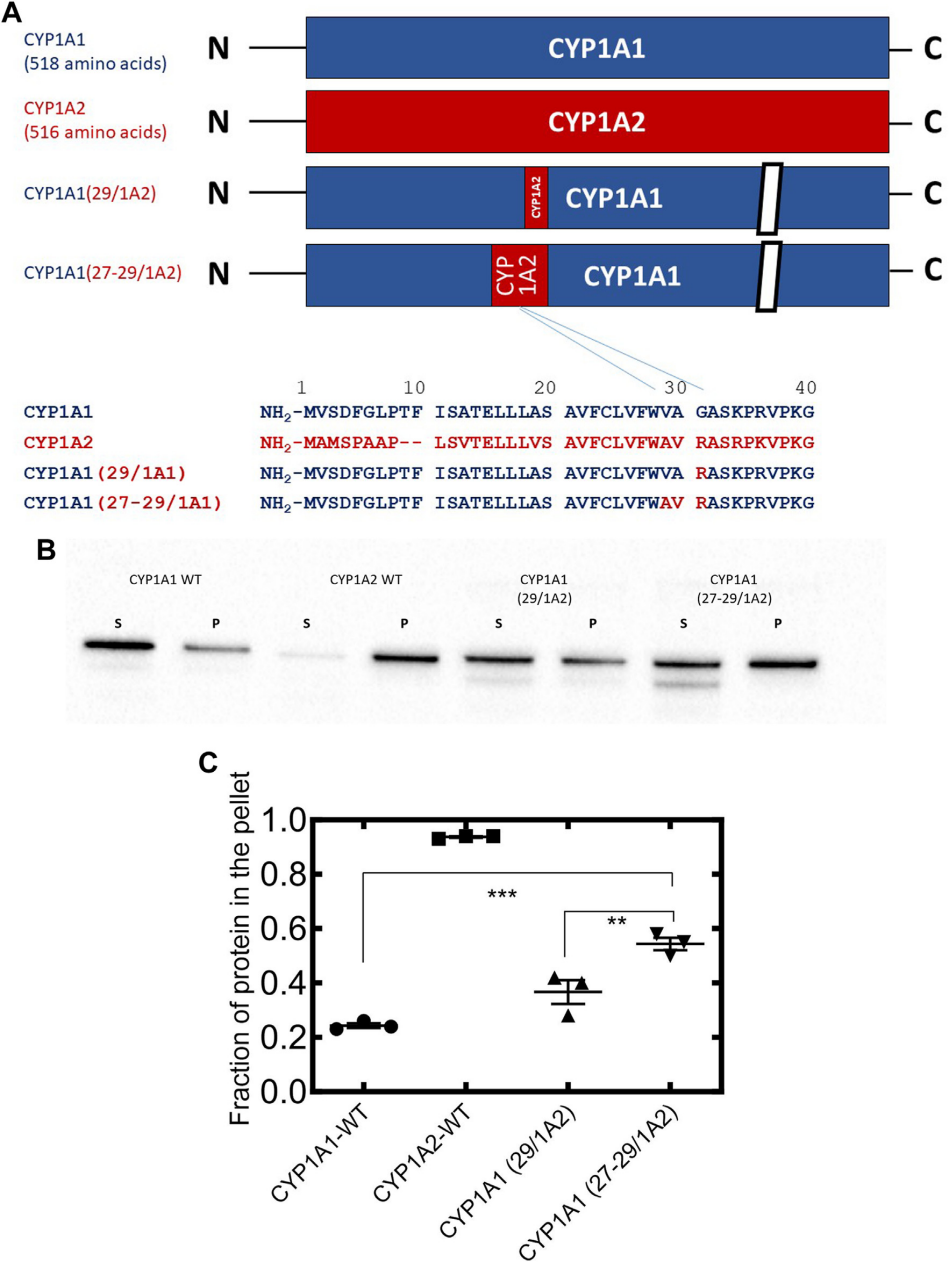
**Effect of N-terminal mutations of CYP1A1 on its microdomain localization—WTCYP1A forms and the chimeras, CYP1A1(29/1A2) and CYP1A1(27-29/1A2).** Membrane localization of WT and chimeric CYP1A forms with C-terminal GFP-tags was determined after transfection of cDNA in HEK cells by Brij 98-based solubilization of PNS (see Experimental procedures section). Other than showing the results using different chimeric CYP1A forms shown, the legend to the figure is as described for Figure 2 for panels *A*–*C*. cDNA, complementary DNA; HEK, human embryonic kidney; PNS, postnuclear supernatant. Statistics were performed using a one-way ANOVA and Bonferroni’s multiple comparison test (∗∗*p* < 0.01; and ∗∗∗*p* < 0.001).

If insertion of the CYP1A1 tripeptide (VAG) into CYP1A2 caused partial relocation of the protein to the disordered regions of the membrane, the reciprocal substitution of the CYP1A2 residues (AVR) into positions 29–31 of CYP1A1 should cause partial relocation of the chimera to the ordered lipid microdomains. This was indeed the case (Fig. 4) as the chimeric protein with the substituted tripeptide from CYP1A2 had roughly 50% of protein in the detergent-resistant pellet compared to only about 25% of WT CYP1A1 being isolated in the pellet after Brij solubilization. In this case, the mutant CYP1A1 with the substituted CYP1A2 tripeptide also was significantly different in microdomain localization from the mutant enzyme with the single R to G substitution at position 31. Furthermore, the single substitution did not have a significantly different localization than that of WT CYP1A1, suggesting again that the tripeptide, and not simply the G/R substitution, was the critical motif in affecting microdomain preference.

### MD simulation

**Figure 5:**
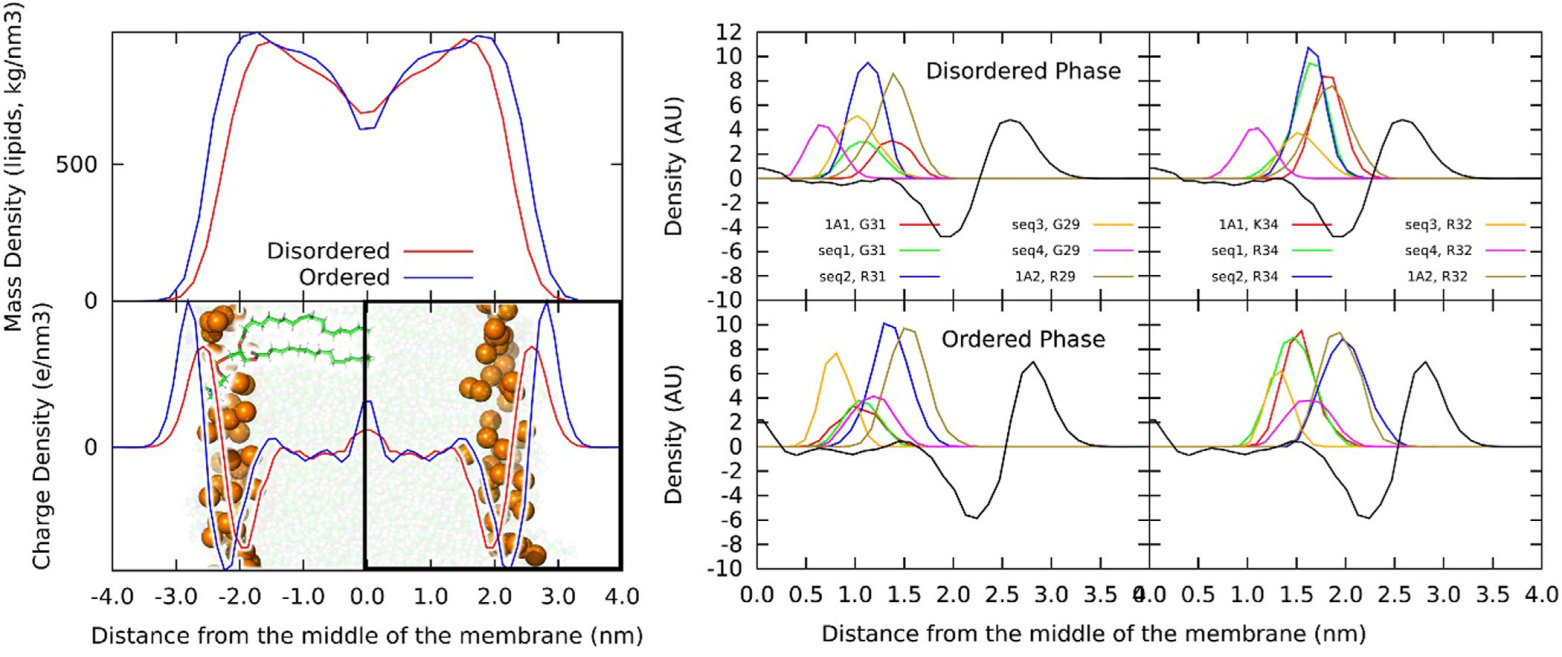
**CYP1A molecular structure and membrane orientation.***Top left*: structure of CYP1A1 immersed in lipid membrane with annotated individual structural features. *Bottom left*: charge density of L_d_ and L_o_ membrane with a negative peak corresponding to the position of phosphates and a positive peak at the position of cholines. A structure of a membrane is used as a background, with *orange balls* showing phosphates and *green* and *white* semitransparent balls representing other lipid atoms. Water is omitted for clarity. The *yellow rectangle* shows a membrane leaflet displayed in the *right panel*. *Right*: mass densities of LYS and ARG 34/32 with respect to the charge density of a membrane leaflet (*black curve*). In L_o_ phase, positively charged R34 in CYP1A2(1-28/1A1) and R32 in CYP1A2 resided at the position of negative charge in lipid membrane which favored their interaction. Ld, liquid disordered membrane regions; Lo, liquid ordered membrane regions.

In order to investigate these chimeras at the atomistic level, a series of MD simulations was carried out (for simulation details, see Supporting information). Prior to simulations with enzymes, two pure membrane models of lipid bilayers were created with a composition mimicking the liquid ordered (L_o_) and liquid disordered (L_d_) domains and equilibrated by MD simulations. As expected, these lipid bilayers showed distinct structural differences corresponding to L_d_ and L_o_ phases according to the lipid compositions (Fig. S3). There were two major differences when comparing the L_d_ and L_o_ membranes. The L_o_ membranes were about 4 Å thicker than the L_d_ membranes (44.8 *versus* 40.2 Å, for L_o_ and L_d_, respectively). Additionally, the L_d_ membranes exhibited a decrease in the deuterium order parameter along the whole oleoyl and palmitoyl chains, corresponding to known behavior of L_d_ and L_o_ phases (Fig. S3) (25). The full-length and chimeric P450 proteins were immersed into both membrane models (Figs. 5 *upper left* and S4) and underwent up to 50-ns MD simulations. The catalytic domain and the N-terminal anchor moved independently of each other because they are interconnected *via* a flexible water-exposed linker region. The P450 catalytic domains lied on the membrane surface regardless of the specific chimera or membrane phase (Fig. S4). Therefore, we focused on the transmembrane helix (TMH) including the mutation sites.

A total of 1.1 μs of atomistic and 200 μs of coarse-grained simulations were accumulated for N-terminal anchors containing the first 52/54 amino acids of P450s sequence in lipid membranes. All 12 mutant-membrane systems were modeled in both atomistic and coarse-grained resolutions, and the detailed analyses can be found in the supporting information. TMHs preserved their α-helical structures and did not display any significant changes in immersion depths in membranes (Fig. S5). In L_d_ phase, TMH orientation was more variable than in rigid L_o_ phase (Fig. S6). TMH-membrane interactions were also affected by point mutations *via* electrostatic interactions due to different localization of charged amino acid residues (Tables S3 and S4 and Fig. S7). When the five proteins CYP1A1, CYP1A2, CYP1A2(1-31/1A1), CYP1A2(1-28/1A1), and CYP1A2(29-31/1A1) were simulated within the L_d_, a positive charge density in the protein was observed at the same membrane depth as the L_d_ negative charge densities, suggesting at least a partial affinity of each of these proteins for the disordered region (Figs. 5 and S7). Conversely, when the same proteins were simulated within L_o_, CYP1A2 and CYP1A2(1-28/1A1) were the only proteins with marked positive charge densities at the same depth as the negative charge density of the membrane (Figs. 5 and S7). Compared to the L_d_ membrane, L_o_ is thicker and has more space between the membrane center and phosphate heads (Fig. 5 *right panel*). Therefore, L_o_ provides a better spatial fit for the positive charge densities of CYP1A2 and CYP1A2(1-28/1A1). The electrostatic interaction of oppositely charged species could explain the preferential affinity of CYP1A1 to the L_d_ and reduced affinity of other chimeras but CYP1A2(1-28/1A1) to L_o_.

### Microdomain localization of NADPH cytochrome P450 reductase

**Figure 6:**
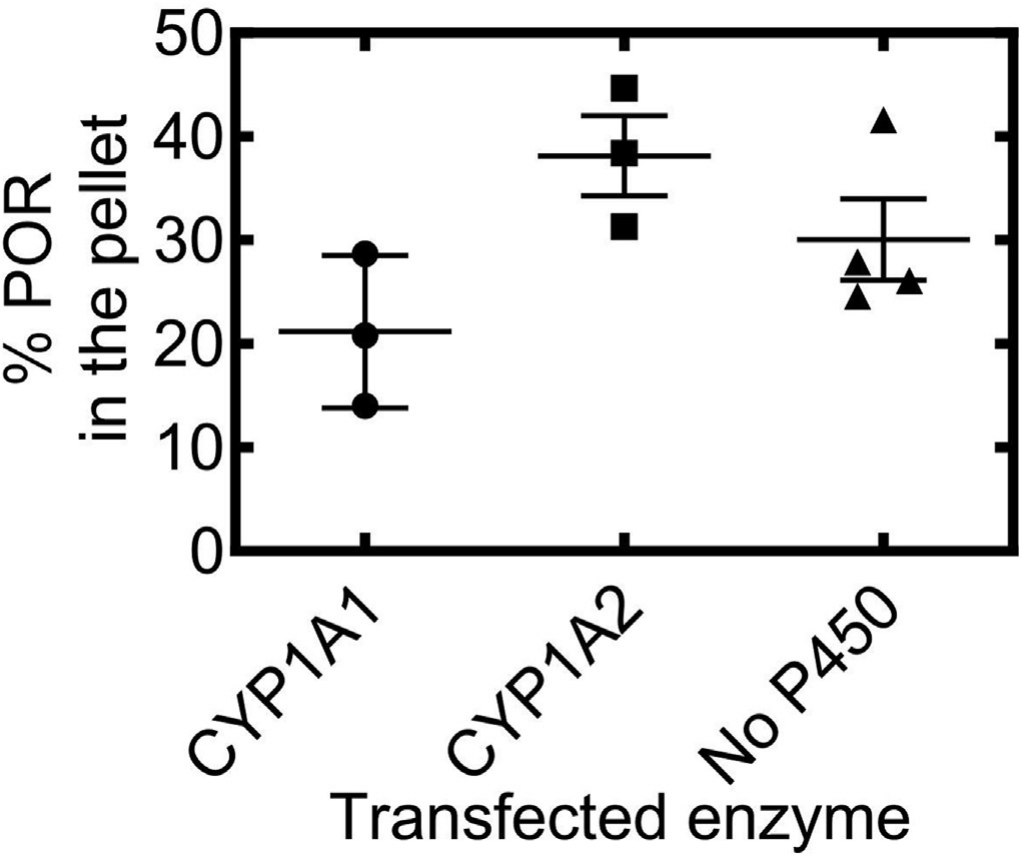
**Percentages of total POR distributed in the Brij 98-resistant pellet after solubilization and ultracentrifugation of PNS from HEK cells in the absence and presence of WT CYP1A1 and CYP1A2.** Each data point is the result from a different transfection experiment involving four 100 mm plates of HEK cells as described in the Experimental procedures section. HEK, human embryonic kidney; POR, NADPH cytochrome P450 reductase; PNS, postnuclear supernatant.

The microdomain localization of the POR was assessed in the presence of heterologously expressed WT CYP1A forms and when no P450 was coexpressed in the HEK 293T/17 cells (Fig. 6). POR expression was relatively unaffected by transfection of P450 complementary DNA and was roughly 1/20th of the P450 expression under the experimental conditions used. The percentage of POR that partitioned to ordered lipid microdomains averaged 29.9% for 14 total experiments with and without CYP1A expression and ranged from 14 to 45% (SD of 8.8%) for the data represented in Figure 6. The localization of POR to ordered lipid microdomains was not significantly influenced by coexpression of the WT CYP1A. Thus, the data do not show a dramatic influence of P450 on POR microdomain localization.

### Effect of substitution of residues 29–31 from CYP1A1 into CYP1A2 on catalytic activity

**Figure 7:**
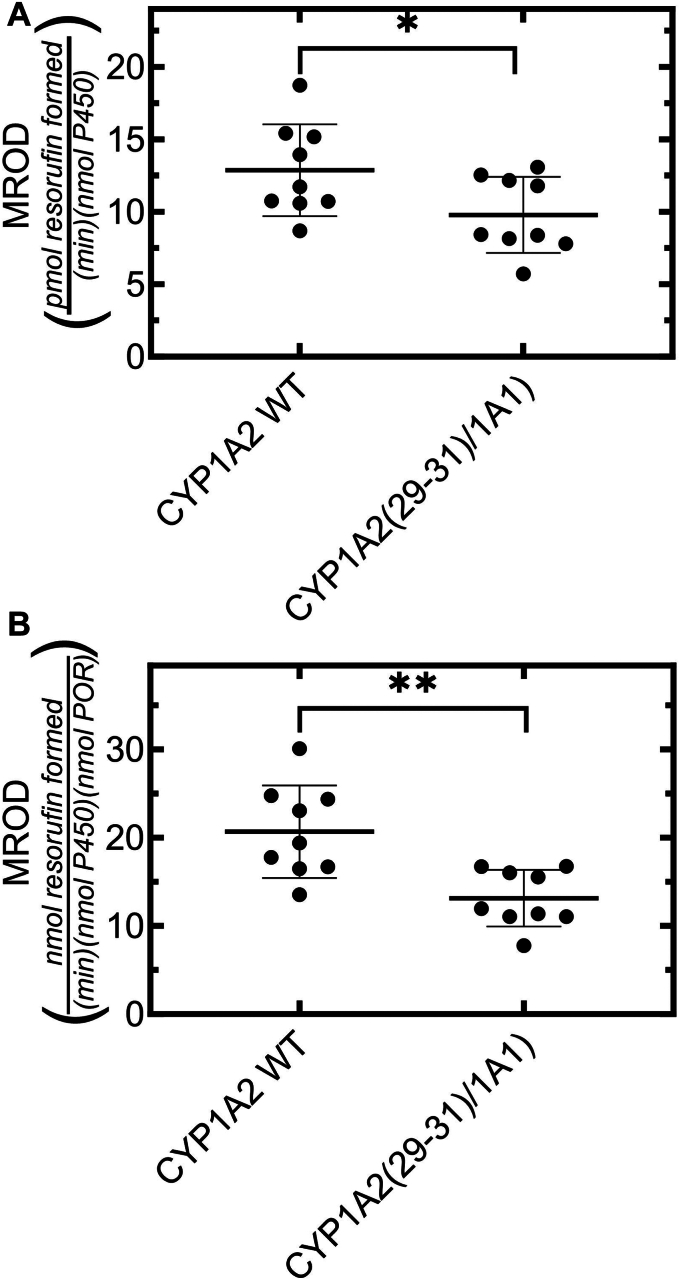
**Rates of MROD catalyzed by HEK cell PNS after expression of WT CYP1A2 and the CYP1A2(29-31/1A1) chimera.** P450s were expressed in HEK cells and PNS were prepared, and MROD was measured by real time fluorescence after addition of a mixture of NADP and 7-methoxyresorufin at 25 °C as described in the Experimental procedures section. The data points were generated from replicate PNS samples prepared from four 100 mm plates each of HEK cells transfected with the indicated P450. Activities were expressed per nmol of the P450 protein used (*A*), and normalized for differences in the endogenously expressed [POR] (*B*). Statistics were performed using an unpaired Student’s *t* test (*p* < 0.05 and ∗∗*p* < 0.01). HEK, human embryonic kidney; MROD, 7-methoxyresorufin-O-dealkylation; PNS, postnuclear supernatant; POR, NADPH cytochrome P450 reductase.

With the transfection of modified CYP1A2s into the HEK 293T/17, it was important to determine that the amino acid substitutions did not lead to a protein that was no longer functional. To accomplish this, 7-methoxyresorufin demethylation was examined for both WTCYP1A2 and CYP1A2 (29-31/1A1). Unlike our previous studies where we cotransfected POR along with the P450 to maintain saturating POR, in the current experiments, we relied on the low levels of endogenous POR within the HEK cells (6). As shown in Figure 7, the activity of the modified CYP1A2 (29-31/1A1) was diminished by about 25% when expressed simply with regard to the P450 concentration (Fig. 7*A*), or when normalized to the concentration of endogenous POR (Fig. 7*B*), showing that the modified construct retained its ability to metabolize 7-methoxyresorufin. These results are similar to our previous findings when comparing the rates of 7-methoxyresorufin-O-dealkylation (MROD) for WT CYP1A2 and a mutant with the first 109 amino acids of CYP1A1 substituted for the corresponding amino acids of CYP1A2 (6). We cannot distinguish whether this decrease was the result of a (1) change in the structure of the protein, or (2) a change in its microdomain localization causing a change in POR availability or affinity; however, the results clearly establish that this mutant CYP1A2 is functionally active.

### Effect of substitution of residues 27–29 of CYP1A2 into CYP1A1 on catalytic activity

In the reciprocal experiment, we substituted residues 27–29 of CYP1A2 into CYP1A1 (*i.e.* CYP1A1 (27-29/1A2) and compared its activity to WTCYP1A1. To accomplish this, we compared the ability of the mutant CYP1A1 and WTCYP1A1 to metabolize 7-ethoxyresorufin. Again, the mutant proteins were less effective catalysts of EROD function when compared to the WT proteins. It is interesting to note that the changes in CYP1A1 activity were greater than what we found when CYP1A2 was mutated, and that these were similar to the larger effects seen on microdomain localization (see Figs. 3 and 4). However, despite the larger effects on activity, we cannot distinguish between changes in microdomain localization or a change in the structure of the protein, but can state that the amino acid (27-29/1A2) substitution did not cause a major change in folding that led to an inactive enzyme.

## Discussion

In the present study, an amino acid motif, at least partially responsible for targeting of CYP1A forms to specific lipid microdomains, was identified. For the first 109 amino acids of CYP1A1 and CYP1A2, the majority of the sequence differences occur within the first 30 positions. Comparison of the membrane microdomain localization of the CYP1A2 (1-28/1A1) and CYP1A2 (1-31/1A1) chimeras (Fig. 2), suggested that the key microdomain-targeting motif was encompassed by the 29-31 amino acids of CYP1A2. This was indeed confirmed by the generation of both the CYP1A2 (29-31/1A1) (Fig. 3) which showed the same relative microdomain distribution as the CYP1A2 (1-31/1A1) chimera (Fig. 2), and the reciprocal CYP1A1 (27-29/1A2) chimera (Fig. 4) which showed significantly higher distribution to ordered lipid microdomains than observed with WT CYP1A1. Furthermore, the amounts of change in the microdomain distribution of the two chimeras with the three amino acids substituted from the alternate CYP1A form were approximately equal suggesting this was the major N-terminal motif for membrane localization. The data also clearly show that the single point mutation involving the G/R transition in this tripeptide motif was not sufficient to achieve a significant change in the microdomain localization of the CYP1A1 and CYP1A2 chimeras. This is supportive of the molecular modeling showing that the single point mutations do not achieve dramatic changes in charge-pairing of lipid phosphate groups with the polar linker region of CYP1A2 in the ordered lipid membrane. To determine whether there were other “latent motifs” that perhaps compete with one another in the N terminal 28 amino acids of CYP1A2, other chimeras including CYP1A2 (1-5/1A1), CYP1A2 (5-8/1A1), CYP1A2 (5-8,29-31/1A1), and CYP1A2 (13,29-31/1A1) were also generated in HEK cells. None of these other chimeras showed significant changes in microdomain localization from that observed for WT CYP1A2 (Fig. S2), further, supporting the important role of amino acids, 27–29, in targeting the enzyme to the ordered microdomain.

**Figure 8:**
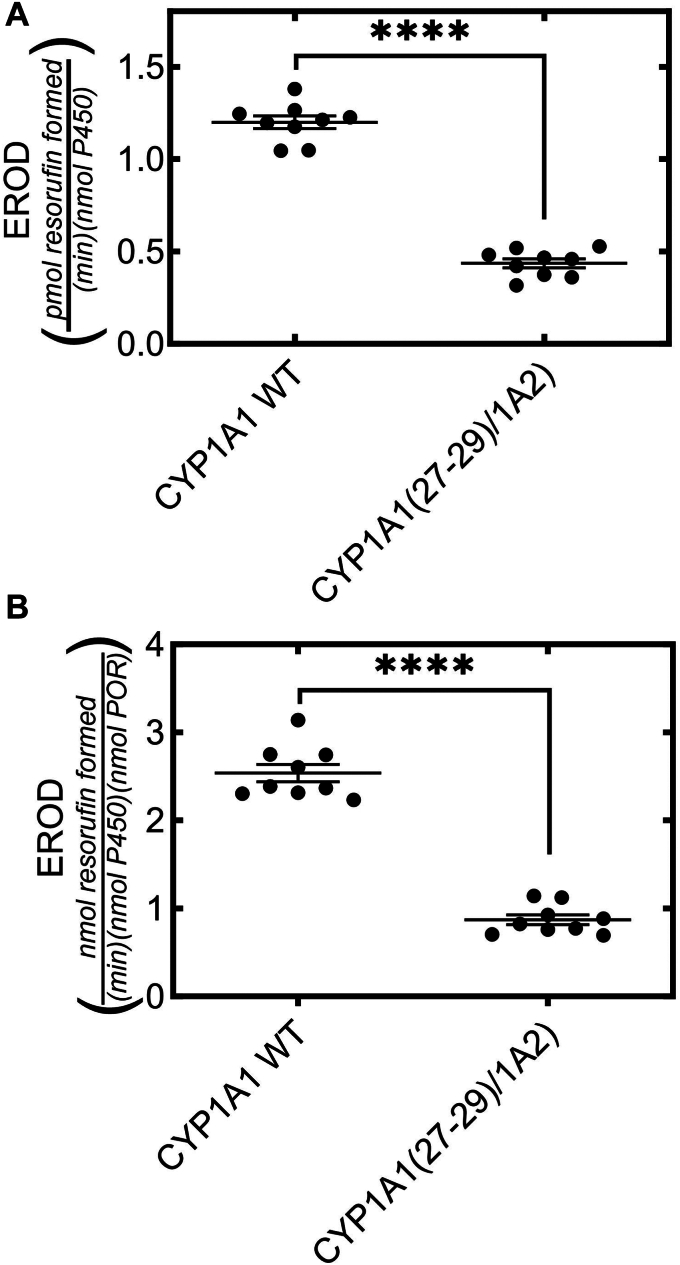
**Rates of EROD catalyzed by HEK cell PNS after expression of WT CYP1A1 and the CYP1A1(27-29/1A2) chimera.** P450s were expressed in HEK cells and PNS were prepared, and EROD was measured by real time fluorescence after addition of a mixture of NADP and 77-ethoxyresorufin at 25 °C as described in the Experimental procedures section. The data points were generated from replicate PNS samples prepared from four 100 mm plates each of HEK cells transfected with the indicated P450. Activities were expressed per nmol of the P450 protein used (*A*), and normalized for differences in the endogenously expressed [POR] (*B*). Statistics were performed using an unpaired Student’s *t* test (∗∗∗∗*p* < 0.0001). HEK, human embryonic kidney; PNS, postnuclear supernatant; POR, NADPH cytochrome P450 reductase.

As mentioned, the activities for each of the expressed constructs tested were able to interact with the endogenous POR and be functionally active (Figs. 7 and 8). However, in each case, the CYP1A2 (29-31/1A1) and CYP1A1 (27-29/1A2) mutants were less active. Although it is tempting to associate these changes with modifications in their microdomain localization, there are several potential reasons for the lower activities. First, the modifications could be affecting the microdomain localization of the proteins. Second, the modifications could influence the ability of the mutant P450s to interact with POR, either by directly affecting POR•P450 complex formation, or by affecting the orientation of the two proteins in the membrane. A third possibility is that the modifications cause a subtle change in the active site of the P450s and lead to diminished activity. Although, we cannot distinguish among the possibilities at this time, we can clearly state that these mutated proteins are active.

The all-atom simulations supported the view that the fit of CYP1A2 into the L_o_ membranes was more energetically favorable when compared to CYP1A1. This may be due, in part, to the basic amino acid at position 29 in CYP1A2, shifting the positively charged amino acid at position 32/34 to associate with the negative charges of phospholipid head groups and providing a primary point for orienting CYP1A2 in the membrane. The 4 Å increase in membrane width of the L_o_ membrane provides additional space to accommodate this protein with the position of positively charged residues matching the positions of negatively charged phosphates. In contrast, lack of the basic amino acid in CYP1A1 and the chimeras (except for CYP1A2 (1-28/1A1) reduces the affinity to the L_o_ membranes.

In this study, we also monitored the lipid microdomain localization of POR and the influence of P450 expression on the distribution of the protein between ordered and disordered lipid regions. HEK cells are ideal systems to ascertain the effects of P450s on POR localization because the cells do not express detectable levels of endogenous P450s. In the frozen cell aliquot used to assess the effect of P450 on POR localization, neither CYP1A1 nor CYP1A2 significantly influenced the microdomain distribution of POR (Fig. 6). POR localization was also assessed in two other frozen cell aliquots when either CYP1A1 or CYP1A2 was expressed, and the POR distributions in the cells were almost identical when the two P450s were compared side by side.

Like other membranous organelles, the ER membrane possesses ordered and disordered microdomains, each having unique lipid compositions (18). Specifically, ER detergent-resistant ordered regions are enriched in sphingomyelin and cholesterol, whereas disordered regions possess a higher relative abundance of phosphatidylcholine and phospholipids with unsaturated fatty acid acyl chains (17). The presence of phospholipids is an important factor affecting function of the microsomal P450 system, and catalytic activity can be detected in reconstituted systems using the appropriate lipid species (26, 27, 28). Alterations in the lipid composition leading to increased fluidity of the membrane were shown to increase normal P450 catalytic activities, underlining the importance of lipid composition in the normal function of these enzymes (29).

There is evidence that N-terminal signal sequences are important in phosphorylation of downstream P450 residues, thus promoting catalytic activity. One study showed that mitochondrial-targeted CYP2E1 possesses an intact N terminus, and that mitochondrial CYP2E1 is phosphorylated at higher levels compared to microsomal CYP2E1 (30). In a subsequent article, the investigators demonstrated that the N terminal 30 amino acids contribute to both mitochondrial targeting and downstream phosphorylation of CYP2E1 (31). The same study reported that this signal sequence is also essential for interaction of CYP2E1 with numerous mitochondrial electron transport proteins. Interestingly, a previous study suggested that N-truncation (Δ2-29) resulted in targeting of the shortened form to the mitochondria (32). Thus, targeting of P450s to organelles other than the ER is not well understood.

It has long been known that amino acid motifs in N terminal or internal regions were necessary for protein trafficking (33, 34). For many P450 species, amino acid motifs target proteins to specific organelles, including the ER (35, 36). Several P450s, including CYP1A1, are bimodally targeted to ER and mitochondrial membranes by N-terminal residues (37). Regarding CYP1A1, one report found that the hydrophilic residues present at positions 34 and 39 were necessary for mitochondrial targeting. The importance of these amino acids in CYP1A2 trafficking was not investigated (38), although the only sequence difference between the forms is the conservative Arg to Lys substitution at position 34. Whether microdomain localization plays a role in intracellular targeting of the P450 enzymes will require further investigation.

Many transmembrane proteins preferentially reside in specific microdomains (16). Ordered regions are vital to the function of numerous proteins including ER and mitochondrial σ-1 receptors, erlin proteins, and prohibitin proteins (39, 40). The presence of ordered microdomains is also essential for assembly, activation, or folding of infectious disease agents including the hepatitis C virus, Shiga-like toxin, and PrP prion (41, 42, 43). Several immune proteins, such as Src homology 2-containing phosphatase 1 and sorbin, are targeted to the plasma membrane ordered regions by critical amino acid sequences in the N or C terminus (44, 45, 46). However, to our knowledge, this is the first study to identify an amino acid motif that targets cytochromes P450 proteins to different regions in the ER membrane.

The most well-studied, general raft-targeting motif is protein palmitoylation (47, 48, 49, 50, 51). Specific raft-targeting transmembrane domain sequences of proteins are not well known. Examples of short (5–10 amino acid) lipid raft-targeting motifs have been reported for immunologic proteins, but no prior research has reported such a motif in P450 proteins. For the tyrosine phosphatase, Src, this involves the six amino acid motif, SKHKED, in the C terminus of the protein (44). In addition, the transmembrane domains of CD44/CD40, and a four peptide motif (FWLY) in Epstein Barr virus latent membrane protein (LMP-1) are required for raft-targeting (52, 53, 54). Previously, we have shown that microsomal CYP2E1 resides in disordered regions of the ER membrane (21). It is conceivable that the amino acid motif involved in microdomain targeting also promotes phosphorylation of downstream residues. Further studies are necessary to determine whether the ER- and mitochondrial-targeting signals possess a disordered-region targeting motif, and more generally, to assess whether protein modifications (such as phosphorylation) influence microdomain localization or affect targeting to other organelles.

Although we examined the potential for the CYP1A microdomain localization motif to be common to other P450 enzymes, none were readily apparent. This was done by examining the sequences for several P450 enzymes CYP1A2, CYP2D1, and CYP2D3 (which reside in ordered microdomains), and CYP1A1, CYP2E1, and CYP3A4 (which reside in the disordered regions) (22, 23). We looked for the presence of the AVR and VAG sequences in the N terminus. We also looked at differences in length of the N terminus, prior to the presence of either a lysine or arginine that would likely interact with the polar head groups of the phospholipids. In none of the cases were common motifs detected that would relate to localization to a specific microdomain. There may be more subtle differences that may affect localization; however, at this time a motif common to other P450 enzymes has not been identified.

Although the functional significance of microdomain localization of the drug metabolizing enzymes is currently not known, it is possible that segregation into different microdomains may promote interactions of proteins into complexes that influence protein function. The concept of organization of plant and animal proteins into the metabolon that facilitates the interaction of proteins into functional complexes was hypothesized in the 1970s (55). Organization of proteins into dynamic metabolons can facilitate the metabolism of substrates by increasing the local substrate concentrations, leading to the sequential metabolism of substrates and their metabolites. Complex formation among many of the drug metabolizing enzymes have been reported, including those between different P450 enzymes (6, 56, 57, 58, 59, 60, 61, 62, 63, 64, 65, 66, 67, 68, 69, 70, 71, 72), as well as P450s with other drug metabolizing enzymes such as UDP-glucuronosyltransferase (73, 74, 75, 76, 77, 78, 79, 80), epoxide hydrolase (73, 81, 82), and heme oxygenase-1 (83, 84, 85). We recently reported that CYP1A2 and CYP2D from the rat liver as well as UDP-glucuronosyltransferases 1A1 and 1A6 all localize within the ordered microdomains (22) with most of the other drug metabolizing enzymes residing predominantly in the disordered regions. Based on several proteomic studies (22, 23, 82), many protein-protein interactions occur between proteins that reside in the same microdomains; however, their localization in different membrane regions does not appear to preclude their interaction. For example, a functional interaction between CYP3A4 and UDP-glucuronosyltransferase 2B7 has been reported (86). Albeit with rat liver, these proteins (CYP3A and UDP-glucuronosyltransferases from the 2B family) were both shown to localize in the disordered membrane regions (23). However, proteins that reside in different microdomains, such as CYP2D6/CYP2E1 (22, 71) and CYP1A2/CYP2E1 (60), have also been shown to form complexes. This could be due to the transitory nature of lipid microdomains (87), or the potential for these complexes to occur at the ordered/disordered membranes interfaces. Further studies will be required to clarify the role of microdomain localization on protein function and the potential for metabolome formation.

In conclusion, this study has refined prior knowledge to show that three specific amino acids are sufficient to affect lipid microdomain localization of CYP1A proteins, suggesting that these residues act as a novel microdomain-targeting motif. Subsequent studies can assess whether these amino acids and other CYP1A internal region domains (*e.g.* the F/G loop) enhance affinity for specific lipid microdomains, and whether microdomain localization is related to targeting into other organelles.

## Experimental procedures

### Materials

The anti-CYP1A1/1A2 antibody used in Brij 98 experiments for all WT and chimeric P450s was purchased from Abcam (ab4227). The GFP primary antibody was from Millipore-Sigma (MAB3580). The anti-POR antibody was from Enzo (ADI-OSA-300-F). The QuikChange II XL Site-Directed Mutagenesis Kit was purchased from Agilent Technologies. Cloning reagents, Lipofectamine 2000, and Dulbecco’s modified Eagle’s medium (DMEM), and PBS were supplied by Invitrogen. P450 sequences were cloned into the pGFP^2-N2^ vector from Bio-Signal Packard. For chimeras over five amino acids in length, restriction enzymes and POR supplies were from New England Biolabs. PCR product was extracted using the QIAquick gel extraction kit (Qiagen). Brij 98 was sourced from Sigma-Aldrich. Resorufin for enzyme activity assays were from AnaSpec.

### Cell culture

Human embryonic kidney (HEK 293T/17 cells were supplied by the American Type Culture Collection (ATCC)). Cells were maintained at 5% CO_2_ and 37 °C throughout growth. The medium was DMEM supplemented with 10% (v/v) fetal bovine serum (Invitrogen) and 1× antibiotic-antimycotic (Gibco-Thermo). Cells were passed up to 25 times from the original stock using 0.5% trypsin (Gibco).

### Cloning of chimeric CYP1A1 and CYP1A2

For generation of chimeras with substitutions over five amino acids in length, overhang PCR was used. This method has been described at length in prior publications (6). Briefly, this approach used two consecutive PCR reactions. First, the desired fragment of CYP1A1 was amplified using a flanking primer against the N terminus of CYP1A1 and an internal cut site primer with an overhang complementary to CYP1A2. The reaction mixture for this step was a 50 μl solution comprised of 200 ng template, 1 mM dNTP, 1 μM each primer, 2.5 U *Pfu* polymerase, and 1× polymerase buffer (Stratagene). The PCR conditions were 40 cycles of denaturation (20 s at 95 °C), annealing (20 s at 59 °C), and extension (45 s at 72 °C), followed by a final three minute extension step. PCR products were separated using a 1% (w/v) agarose gel and extracted using the QIAquick extraction kit. The second PCR incorporated the copied CYP1A1 fragment from the first step into the desired site on CYP1A2 using the primer overhang sequences, generating a CYP1A1-1A2 chimeric protein (*i.e.* with the N terminus from CYP1A1 and the downstream sequence from CYP1A2). The construct was identified and extracted using agarose gel and the QIAquick extraction kit.

### CYP1A transfection of HEK 293T/17 cells

Each transfection condition was performed using four, 20 × 100 mm tissue culture plates using 4 to 5 μg of CYP1A expression plasmid DNA per plate. The transfection protocol followed manufacturer specifications for Lipofectamine 2000 with the following exceptions. Three microliters of lipofectamine was used for every microgram of plasmid DNA that was to be used for the experiment. The plasmid with P450 complementary DNA was diluted into a volume of opti-MEM (Gibco) that was equal to 20 times the volume of lipofectamine used in the experiment. The lipofectamine was then diluted into the same volume of lipofectamine for exactly 5 min. After the five-minute incubation, the lipofectamine was transferred to the tube containing the previously diluted DNA. The mixed solution was then incubated another 20 min before being pipetted two or three times and distributed to the tissue culture plates containing approximately 10 ml of DMEM with HEK 293T/17 cells at 65 to 90% confluence. Cells then incubated in the CO_2_ incubator for 6 h before the media were replaced with 10 ml of sterile-filtered DMEM containing 1 mM aminolevulinic acid. The cells then incubated another 20 to 40 h in the CO_2_ incubator before harvesting and lysing cells to extract the PNS.

### Generation of PNS

To harvest cells post transfection, the media were suctioned off and the cells were collected by repeatedly pipetting with 4 ml of ice-cold PBS (Gibco). The 4 ml volume was used for every two plates, so the final, initial cell suspension was typically 8 ml. The suspended cells were transferred to 15 ml tubes and centrifuged at 1000*g* for 5 min, and washed once with another 8 ml of ice-cold PBS. Following another round of centrifugation, the cell pellet was resuspended in 5 ml of ice-cold hypotonic buffer (10 mM Hepes (pH = 7.9), 10 mM KCl, 1.5 mM MgCl_2,_ 0.5 mM DTT, and 1 mM PMSF (added from a freshly made 100 mM stock solution in isopropyl alcohol)). The cells were then incubated on ice for 20 min. After the incubation, cells were centrifuged, the supernatant was suctioned off, and the cells were then resuspended by gentle pipetting using a large orifice pipette tip in 1 ml of ice-cold hypotonic buffer that was also supplemented with a mini-tablet of Roche EDTA free, protease inhibitor cocktail that was dissolved in 5 ml of hypotonic buffer before adding a 1 ml aliquot to the cell pellet. The cell suspension was subjected to seven passes through a 27½ gauge needle, and the resulting lysate was centrifuged 900*g* for 10 min. The resulting PNS was collected (900–1000 μl) and used for Western blotting, enzymatic assays, or for solubilization with Brij 98.

### Solubilization of PNS with Brij 98

A 1% Brij 98 (Sigma-Aldrich) solution was prepared by taking a 0.1 ml aliquot of Brij 98 (that was previously pipetted (after melting the detergent) to a glass test tube and frozen at −20 °C) and placing it briefly in a beaker of water heated to 55 °C. Within 5 min, the Brij 98 wax melted. Solubilization buffer (50 mM Hepes (pH 7.25), 150 mM NaCl, and 5 mM EDTA), that had been heated for 30 min in a 50 ml conical tube in the same water bath with the test tube of detergent, was added so that the final Brij 98 concentration was 1% (10 ml final volume). Immediately after adding the solubilization buffer, the detergent solution was vortexed at maximum speed until all detergent crystals were dissolved. The 1% Brij solution and the remaining solubilization buffer (≈40 ml) were both immersed on ice for 15 min before 5 ml of 1% Brij 98 solution was transferred to a fresh tube. Another mini tablet of Roche EDTA free, protease inhibitor was added to the 5 ml detergent aliquot, and a complete tablet of Roche protease inhibitor and 350 to 400 μl of 1 mM PMSF (1:100 dilution of a 100 mM PMSF stock solution) were added to the remaining, ice-cold tube of solubilization buffer.

For detergent solubilization, the PNS, prepared as described above, was added as a 0.5 ml aliquot to a clear, 4.5 ml ultracentrifuge tube for a SW55Ti swinging bucket rotor. An equal volume of the 1% Brij 98 solution was added by a repeater pipette, and the tubes were immediately incubated in a water bath at 37 °C for 5 min. After this incubation, the tubes were immediately immersed on ice before 2.0 ml of the ice-cold solubilization buffer with protease inhibitors was added to stabilize the tubes for ultracentrifugation. After balancing the tubes with solubilization buffer, the samples were then centrifuged at 100,000*g* for 1 h at 4 °C. Immediately after ultracentrifugation, supernatants were poured off into fresh 15 ml conical tubes, and the ultracentrifuge tubes with the residual detergent-resistant pellets were immersed in ice. These pellets were then detached from the bottom of the tubes with a glass stir rod and then resuspended in 3 ml of solubilization buffer using five passes in a 5 ml Potter-Elvehjem homogenizer. Aliquots (0.15 ml) of the supernatants, resuspended pellets, and the remaining PNS were boiled for 20 min after an addition of 44 × NuPAGE LDS sample buffer (Invitrogen) supplemented with 1:20 2-mercaptoethanol (Bio-Rad). After boiling, the samples were flash frozen in liquid nitrogen and stored at −80 °C. Storing in this manner minimized proteolytic degradation of the CYP1A forms.

### Western and immunoblot analysis

Solubilized and detergent-resistant extracts and PNS from HEK 293T/17 cells were loaded onto NuPAGE 10% Bis-Tris Gel (Invitrogen) and run at 150 V for approximately 1 h. When enzymatic activities were measured, the levels of enzyme expression were determined using purified, human P450 reductase and purified GFP protein (Millipore) as gel standards. The gels were transferred to nitrocellulose using the Power Blotter system (Thermo Fisher Scientific), blocked for 1 h with PBS containing 0.05% Tween 20 (Bio-Rad) and 2% bovine serum albumin. Primary antibodies (listed in *Reagents*, above) were applied overnight, and secondary horseradish peroxidase conjugated anti-mouse or anti-rabbit antibody was applied for 1 h diluted at 1:4000. Chemiluminescence was activated using the Supersignal West Pico kit (Thermo Fisher Scientific).

### Enzyme activity assays

For each substrate reaction, 43 μl of PNS derived from cellular lysates of HEK 293T cells expressing the indicated WT and chimeric CYP1A forms were mixed with stock solutions of Hepes-NaOH, MgCl_2_, and catalase to final concentrations of 0.05 M, 15 mM, and 275 U/ml, respectively and water to a final volume of 90 μl in triplicate wells of a 90-well plate alongside wells corresponding to a 0.001 to 1 mM resorufin standard curve. To start the reaction, 10 μl of a mixture containing 3.6 mM NADPH and 100 μM 7-methoxyresorufin (for CYP1A2), or 77-ethoxyresorufin (for CYP1A1) was added to each well. The reaction was measured in real time at 25 °C as indicated, by monitoring florescence for 20 min (excitation 535 nm/emission 585 nm) using a SpectraMax M5 plate reader.

### Homology modeling

The all-atom protein models were based on a human CYP1A1 template (88). The CYP1A2/CYP1A1 homology models were created by the Modeller 9.25 plugin in Chimera 1.13.1 (https://www.cgl.ucsf.edu/chimera/cgi-bin/secure/chimera-get.py?file=win64/chimera-1.13-win64.exe). Using the sequences with Uniprot IDs P05176 (rabbit CYP1A1) and P00187 (rabbit CYP1A2) (89, 90). In the case of the tail only sequences (see Table S1), the model was built from the N terminus up to the end of the sequence PGPWGWPL (amino acid 52 in CYP1A1), which is conserved in all three sequences (namely human CYP1A1 template, rabbit CYP1A1, and rabbit CYP1A2). Out of the five models generated by default by Modeller for each case, the one with the closest match of the anchor helix to the template conformation was selected. The models of the chimeric sequences were built from the CYP1A2/CYP1A1 models using the point mutation and editing tools of PyMol (https://pymol.org/) (91). In whole-protein models, the heme molecule was retained in its template position.

### All atom simulations

The protein models were inserted into a liquid ordered (L_o_) and disordered (L_d_) membranes of different lipid compositions using the CHARMM-GUI membrane builder tool (92). For the L_d_ system, a simple mixture of 1-palmitoyl-2-oleoyl-sn-glycero-3-phosphocholine (POPC) and 1-palmitoyl-2-oleoyl-sn-glycero-3-phosphoethanolamine (POPE) (molar ratio 7:3) lipids was used whereas in case of Lo membrane the mixture was enriched by cholesterol and sphingomyelin lipids (molar ratio CHL:POPC:POPE:PSM, 2:4:2:1) to mimic the experimental setup. All systems were solvated using TIP3P water model (93). In order to maintain the physiological environment, 0.154 M Na^+^ Cl^−^ ions were added to the hydrated box. Additionally, we prepared and analyzed pure L_o_ and L_d_ membrane models.

In all-atom simulations CHARMM36 force field parameters for lipids, proteins, and ions were used (94). In order to keep constant temperature at 303 K the Nosé–Hoover thermostat with temperature coupling constant of 1 ps was used (95, 96). The constant pressure of 1 atm was kept by Parrinello-Rahman barostat with semiisotropic conditions, pressure coupling constant of 5 ps and compressibility of 4.5 × 10^−5^ bar^−1^ (97). Long range electrostatics were treated by Particle Mesh Ewald summation up to 1.2 nm (98). Cutoff for short range van der Waals interaction was set at 1.2 nm. Periodic boundary conditions were treated in all directions (99). All systems were simulated using GROMACS 5.0 software (http://ftp.gromacs.org/pub/gromacs/gromacs-2018.1.tar.gz) and 2018.1 version of the respective simulation package. All systems were subject to energy minimization, preequilibration, and the total production time length varies for different systems (see Table S2).

### CG simulations

The coarse-grained (CG) membrane models were built by CHARMM-GUI Martini builder in Martini 2.2 force field (100, 101, 102). The membrane contained 700 and 744 lipids in L_d_ and L_o_ phases, respectively, keeping the same lipid ratio as in all atom simulations. CG membranes were equilibrated with predefined protocol from CHARMM-GUI, and then the production run was performed for 5 μs with temperature kept at 303.15 K and pressure kept at 1.0 bar.

P450 anchor models were converted from the all-atom models using martinize.py script. They were oriented along the z-axis and pasted into the membrane structure (aligning the center of masses of lipids and the anchor). Afterward, we performed energy minimization, gradually increasing the length of the simulation step up to 20 fs and the production run for 10 μs for all mutants and 50 μs for CYP1A1 and CYP1A2 in L_o_ phase and 5, 10, and 20 μs in L_d_ phase (see Table S2).

### Analysis

The majority of analyses were performed using standard GROMACS 5.0 tools. In all atom simulations the anchor tilt angle was analyzed by *gmx helixorient* tool and defined by C_α_ atoms of residues #13 and #25 residing on opposite sides of the helix and membrane normal. Charge density plots of membranes and individual amino acid positions were calculated *via gmx density* tool. The *g_mmpbsa* tool was used to estimate energetic contribution of each anchor residue to total protein-membrane binding energy using Molecular mechanics Poisson-Boltzmann method (103). The average number of hydrogen bonds between protein residue and surrounding waters were evaluated by the *cpptraj* implemented in AMBER tool 19.9 (https://ambermd.org/AmberTools.php), which was used for the definition of the hydrophobic region of TMH (104). The membrane thicknesses were analyzed by VMD MEMBPLUGIN 1.1 (https://www.ks.uiuc.edu/Development/Download/download.cgi?PackageName=VMD) (105).

All experimental data were expressed as the mean ± SD, with statistical significance assessed using a one-way analysis of variance and either Bonferroni’s multiple comparison test, or Student’s *t* test (∗*p* < 0.05; ∗∗*p* < 0.01; ∗∗∗*p* < 0.001; and ∗∗∗∗*p* < 0.0001).

## Data availability

All the data are included in the manuscript and supporting information.

## Supporting information

This article contains supporting information.

## Conflict of interest

The authors declare that they have no conflicts of interest with the contents of this article.
