# Supplementary material for: Identification of the N-terminal residues responsible for the differential microdomain localization of CYP1A1 and CYP1A2
Source: J Biol Chem. 2024 Oct 22;300(11):107891. doi: 10.1016/j.jbc.2024.107891 (PMC11603000; doi:10.1016/j.jbc.2024.107891)
Supplement: Supporting Information [file mmc1.docx]

Fuchs, R.M., Reed, J.R., Connick, J.P., Paloncýová, M., Šrejber, M., Čechová, P., Otyepka, M., Eyer, M.K. and Backes, W.L.

Supporting Information for

Identification of the N-terminal residues responsible for the differential microdomain localization of CYP1A1 and CYP1A2

When determining the localization of CYP1A1 and CYP1A2, we examined relative expression levels of their transfection, and that of endogenous POR. For the experimental results included in this study, four different frozen stocks of HEK cells were used for the various studies involving CYP1A cDNA transfection. The average expression after transfection was 10 to 15 pmol of CYP1A per ml of PNS derived from four, 100 mm plates of HEK cells (Figure S1). Variability in cDNA expression can be attributed to the following: cells were incubated for 20 to 48 hrs after transfection; cell confluence at the time of transfection varied from 70-90%; and 4-5 micrograms of plasmid DNA was used for the transfection experiments. In all experiments comparing the detergent solubilization of specific CYP1A mutants, wild type controls were also expressed under identical conditions in order to confirm that results were not skewed by variabilities in P450 expression. In contrast, endogenous levels of POR were less variable, but were extremely low, averaging about 1 pmol/ml.


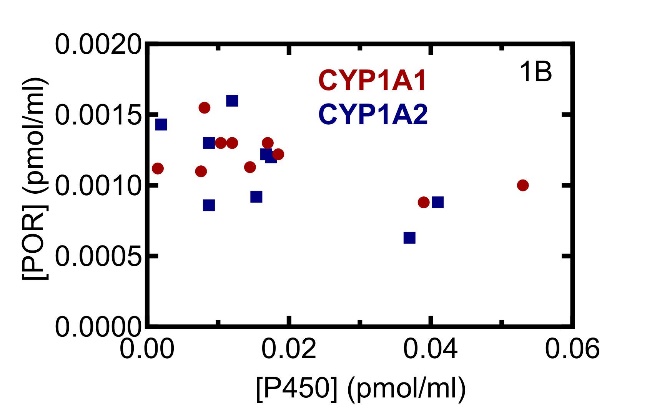

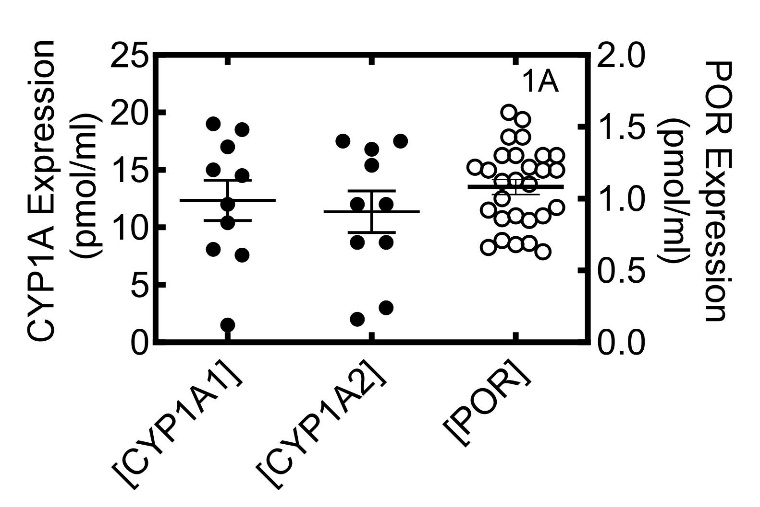


**Fig. S1 – Variability in CYP1A and POR protein expression after transfection of CYP1A DNA.** (A) Transfection of either CYP1A1 or CYP1A2 into HEK293T/17cells led to variability of the P450 levels. When the P450s were transfected, endogenous POR levels were about 10 to 20-fold lower than the P450 levels and also were variable. (B) In this panel, the levels of endogenous POR expression were compared to the transfected levels of CYP1A1 and CYP1A2. These results show that endogenous POR concentrations were not affected by transfected P450 levels.


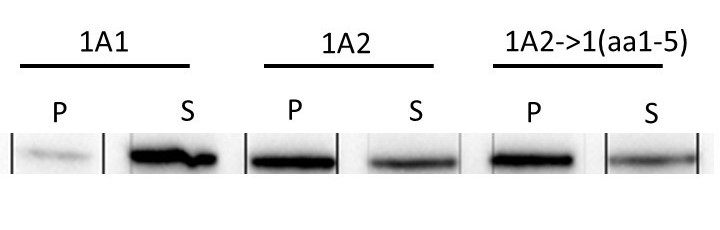


**Fig. S2 – Effect of substitution of CYP1A1 residues 1-5 (MVSDF) into CYP1A2 on its membrane microdomain localization.** Membrane localization of wild type and chimeric CYP1A forms with C-terminal GFP-tags was determined after transfection of cDNA in HEK cells by Brij 98-based solubilization of PNS (see Experimental Section). Other than showing the results using different chimeric CYP1A forms shown, the legend to the figure is as that described for Figure 2.

*Table S1:* ***Sequences used for MD simulations.*** *This table shows the sequences of the proteins used for MD simulations.  The residues in* ***bold*** *represent the residues that were mutated.  The sequences in blue represent CYP1A1, and those in red represent CYP1A2.*

| Model name | N-terminus | Conserved part | Mutation site | Rest of the protein |
| --- | --- | --- | --- | --- |
| CYP1A1 | MVSDFGLPTF I | SATELLLAS AVFCLVFW | VA **G** | AS**K**PRVPKG... |
| CYP1A2(1-31/1A1) | MVSDFGLPTF I | SATELLLAS AVFCLVFW | VA **G** | AS**R**PKVPKG.. |
| CYP1A2(1-28/1A1) | MVSDFGLPTF I | SATELLLAS AVFCLVFW | AV **R** | AS**R**PKVPKG.. |
| CYP1A2(29-31/1A1) | --MAMSPAAP L | SVTELLLVS AVFCLVFW | VA **G** | AS**R**PKVPKG.. |
| CYP1A2(31/1A1) | --MAMSPAAP L | SVTELLLVS AVFCLVFW | AV **G** | AS**R**PKVPKG.. |
| CYP1A2 | --MAMSPAAP L | SVTELLLVS AVFCLVFW | AV **R** | AS**R**PKVPKG.. |

**Table S2.** The total production times of different simulated systems.

| **Model** | **Simulation time - anchor** | | | | **Simulation time – full CYP** | |
| --- | --- | --- | --- | --- | --- | --- |
|  | CG L_o_ (μs) | CG L_d_ (μs) | AA L_o_ (ns) | AA L_d_ (ns) | L_o_ (ns) | L_d_ (ns) |
| CYP1A1 | 50 | 20 | 100 | 80 | 50 | 20 |
| CYP1A2(1-31/1A1) | 20 | 5 | 100 | 80 | 20 | 20 |
| CYP1A2(1-28/1A1) | 10 | 5 | 100 | 80 | 20 | 20 |
| CYP1A2 | 50 | 10 | 100 | 80 | 50 | 30 |
| CYP1A2(29-31/1A1) | 10 | 5 | 100 | 80 | 20 | 20 |
| CYP1A2(31/1A1) | 10 | 5 | 100 | 80 | 20 | 20 |

*All-atom simulations*

During our simulations, we monitored differences in the structure of L_o_ and L_d_ membranes. In agreement with general knowledge, we observed higher ordering of L_o_ membrane in terms of higher values of the deuterium order parameter, a measure which is inversely related to membrane fluidity (Figure S3). Further, L_o_ phase was by ~4 Å thicker than L_d_ phase (44.8 and 40.2 Å for L_o_ and L_d_ phase, respectively) and average area per lipid (over all lipid types) in L_o_ phase was 51 Å^2^, while in L_d_ phase we observed 63 Å^2^. All these observations agree with both theoretical and experimental studies of membrane phase behavior and therefore these two systems provide good models for their comparison.


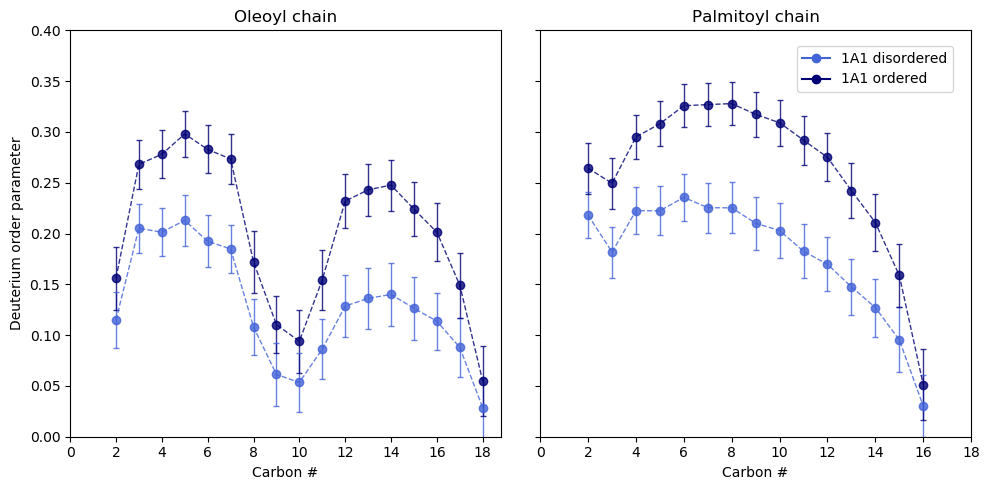


*Figure S3: Deuterium order parameters of palmitoyl and oleoyl acyl chains of the POPC and POPE lipids for L_d_ and L_o_ phase.*

We studied membrane embedded CYPs (full sequence length) for all the combinations of mutants and membrane compositions (Figure S4). The length of these simulations varied from 20 to 50 ns based on studied system; the total production time lengths are stated in Table S2. While the position of the catalytic domain of the protein varied only slightly between individual models, the transmembrane helix (TMH) on the N-terminal side of the protein could adopt much larger number of conformations. The TMH is connected to the catalytic domain of the protein by flexible loop composed of approximately 20 amino acid residues. Therefore, the whole N-terminal anchor of the protein could move independently of the movement of the catalytic domain. Conformational movement of the N-terminal anchor was restricted only by the presence of membrane. In further studies we focused exclusively on the N-terminal anchor.


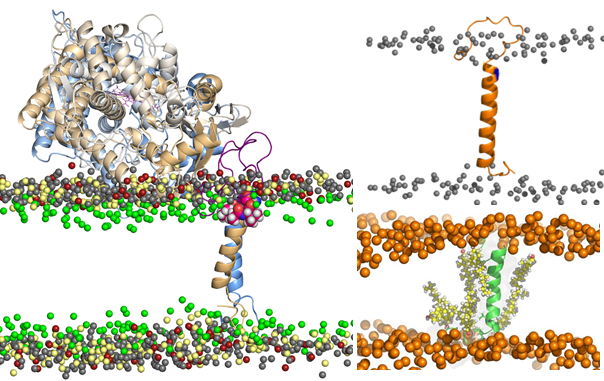


*Figure S4: The overlay of the final structures of the whole-protein simulations of CYP1A1 (blue cartoon) and CYP1A2 (orange cartoon) in the L_o_ membrane. The phospholipid phosphate and cholesterol oxygen atoms are shown as balls (POPC gray, POPE yellow, PSM red, CHOL green). The mutation residues are shown as magenta spheres, and the flexible hinge region is highlighted in purple. The rest of the lipid molecules and waters are omitted for clarity.*

We performed simulations of the N-terminal anchor on models containing only the first 52/54 CYP residues, based on their sequence (Table S1). These models contained simple α-helix surrounded by random coil from both ends embedded into membranes (Figure S5). In all cases, TMH preserved its secondary conformation only with mild differences in its length, affected directly by mutated residues. No significant alterations of the anchor positions were observed in either L_d_ or L_o_ membranes with respect to initial poses. Global differences in membrane properties caused by different phase (such as membrane thickness or area per lipid) did not lead to changes in TMH immersion.


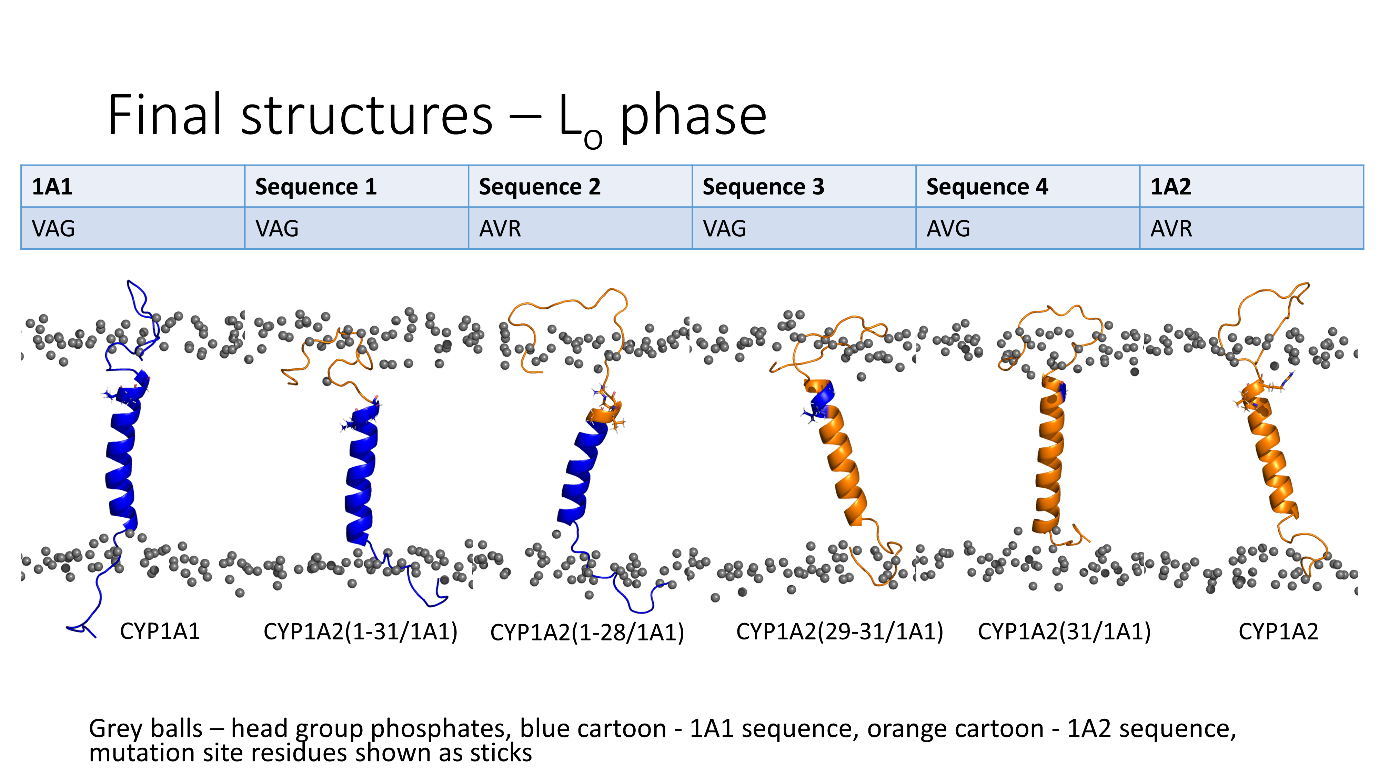


*Figure S5: Final structures of transmembrane helices (TMH) in L_o_ phase. Membrane phosphates are represented as grey balls, TMHs as blue or orange cartoons. Blue color corresponds to wild-type CYP1A1 sequence, orange shows wild-type CYP1A2 sequence. Mutation sites are depicted as sticks. Other lipid tails and waters are omitted for clarity.*

Chimeric TMHs folded the same in ordered and disordered regions, but their orientation versatilities differed across these two phases. In our simulations, the orientation of TMH was more uniform in L_o_ phase than in L_d_ phase. In L_d_ phase we monitored significant fluctuations in angles between TMH axis and the membrane normal with the most noticeable differences for CYP1A2(1-28/1A1) and CYP1A2. The TMH of CYP1A2(1-28/1A1) was oriented almost perpendicularly to the membrane plane (~8 °), while e.g. CYP1A2 TMH was most often tilted by ~24° to the membrane normal with fluctuations up to ~40° (Figure S6). In L_o_ phase the overall distribution of TMHs orientation was narrower, as result of more rigid membrane structure, with all models having the most populated tilt of 14-20°. This is in agreement with the expected higher ordering of L_o_ phase.


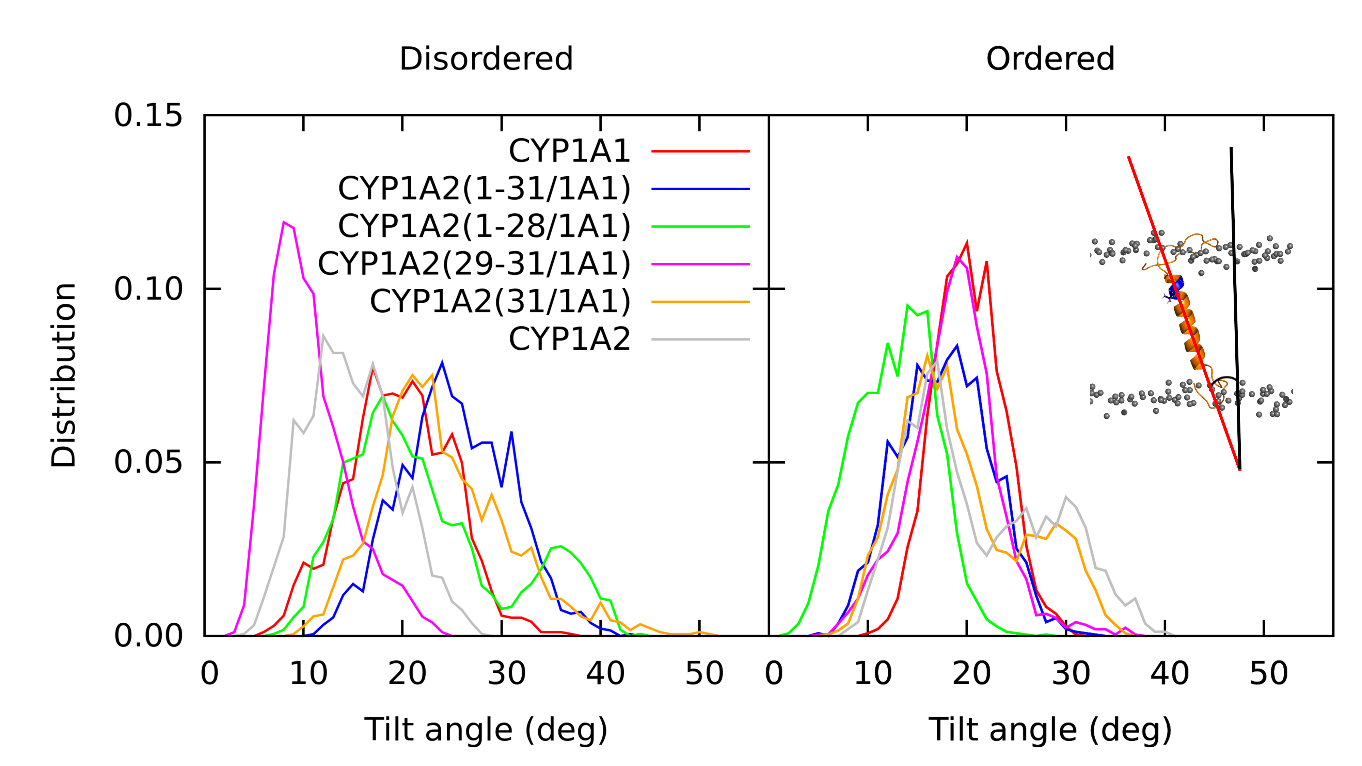


*Figure S6: Transmembrane helix tilt angle in respect to the membrane normal with an inset demonstrating the monitored angle.*

To predict the affinity of each chimera for both phases, interaction energies were calculated for each condition. The interaction energies between TMH residues localized in the hydrophobic region of anchor and membrane showed no noticeable trend among mutant variants (Table S3). It is worth mentioning that only regions without water contact were subjected to analyses, suggesting that differences in membrane-anchor interactions originated from the mutation site located near membrane head group region.

*Table S3: MM/PBSA analysis results of the interaction energies between the hydrophobic region of TMH (between LEU14/16 and TRP26/28) and lipids in L_o_ and L_d_ phase and their differences.*

| kJ/mol | L_o_ | L_d_ | L_d_-L_o_ |
| --- | --- | --- | --- |
| CYP1A1 | -212 ± 32 | -194 ± 33 | 18 |
| CYP1A2(1-31/1A1) | -197 ± 33 | -206 ± 31 | -9 |
| CYP1A2(1-28/1A1) | -192 ± 32 | -182 ± 32 | 10 |
| CYP1A2(29-31/1A1) | -211 ± 34 | -191 ± 33 | 20 |
| CYP1A2(31/1A1) | -177 ± 30 | -184 ± 33 | -7 |
| CYP1A2 | -191 ± 33 | -195 ± 35 | -4 |

A difference in TMH-membrane interaction can lie in the membrane structure and charge localization, as the L_d_ and L_o_ membrane phases differ in membrane thicknesses and therefore also in the charge densities. The charge densities differ both in the relative height (both negative and positive peaks are higher in L_o_ phase than in L_d_ phase) and in the position of the peak corresponding to the lipid head groups (Figure S7). The comparison of positions of the negative peak (corresponding to the position of phosphate groups) with positions of point mutations at G/R 29/31 or K/R 32/34, showed significant differences between both phases. In the L_d_ phase, the relative positions of point mutation site residues 29/31 and K/R 32/34 are conserved across all mutants. However, in the L_o_ phase, the 29-31 mutation (in CYP1A2 and CYP1A2(1-28/1A1)) lead to a slight shift in the residue position toward lipid head groups. This shift was even more profound in case of residue R32/34, where the positions of positively charged amino acids directly overlapped the peak of negative membrane charge density. For the positively charged arginine such interaction is therefore more favorable. We quantified this effect in terms of integral overlaps (both amino acid mass densities in respect to membrane charge densities, as well as directly multiplying charge densities, see Table S4) and we observed that the most favorable interactions with L_o_ phase could be observed in CYP1A2 and CYP1A2(1-28/1A1), while all other mutations significantly decreased the respective electrostatic interactions. The electrostatics favors the interaction of positively charged residues with overall negatively charged membrane head group region and explains the preference for the L_o_ phase.


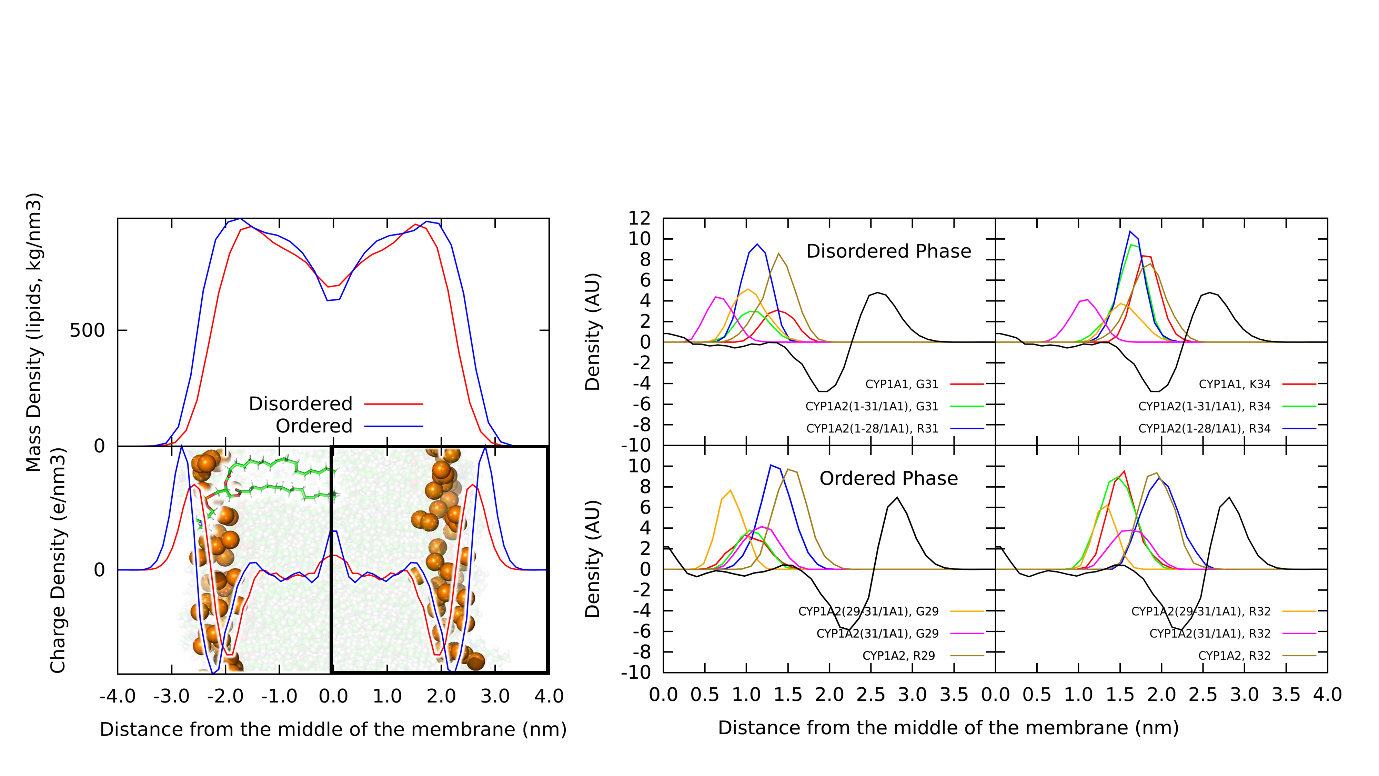


*Figure S7: The mass and charge densities of the membrane systems.*

*Left: The mass (top) and charge (bottom) of the L_o_ and L_d_ phase taken from system with CYP1A1 TMH. A schematic of the membrane position shows the position of the phospholipid phosphate atoms as orange balls, other lipid atoms are depicted as semitransparent green and white balls. Water and TMH is omitted for clarity.*

*Right: A close-up on one membrane leaflet (highlighted as thick black rectangle in the left figure) with mass densities of selected residues and charge density of the membrane (black curve). L_d_ phase is shown in the top panel, L_o_ phase is in the bottom panel. The mass and charge densities are rescaled in order to fit into the figure and show relative positions of charged amino acid residues and charges in lipid membrane. The pictured residues G29/R29/G31 (left panel) and R32/R34/K34 (right panel) occupy corresponding position in the protein structure but have different positions in the sequence, due to the two residue difference between the CYP1A1 and CYP1A2 homologues and the corresponding mutants derived from them. The G29/R29/G31 residue is a part of the examined point-mutation site.*

*Table S4: Integrals of the overlaps of mass densities of individual positively charged residues with a charge density of the membrane and integral of charge densities of amino acid residues and lipid membrane.*

|  |  | Mass-charge integral (AU) | | Charge-charge integral (AU) | |
| --- | --- | --- | --- | --- | --- |
| Mutant | Res# | L_d_ | L_o_ | L_d_ | L_o_ |
| CYP1A1 | K34 | 12.10 | 0.22 | 5.04 | 1.46 |
| CYP1A2(1-31/1A1) | R34 | 9.06 | -0.11 | 3.62 | -0.71 |
| CYP1A2(1-28/1A1) | R34 | 8.94 | 12.72 | 3.12 | 3.13 |
| CYP1A2(29-31/1A1) | R32 | 3.26 | -0.24 | -0.76 | 0.24 |
| CYP1A2(31/1A1) | R32 | 0.52 | 1.07 | -0.05 | -0.59 |
| CYP1A2 | R32 | 15.02 | 12.82 | 4.82 | 3.44 |

*Coarse-grained simulations*

We performed coarse grained simulations on very long time-scales in order to observe possible accumulation of individual lipid types in proximity of TMH. We simulated CYP1A1 and CYP1A2 TMHs in L_o_ phase for 50 μs and other models for 10 μs and monitored local densities of lipids around TMH. In the L_o_ phase, cholesterol molecules accumulate around the protein anchor (Figure S8), which was consistent for all models over the whole length of the μs-scale coarse-grained simulations and does not seem to be affected by any studied mutation.


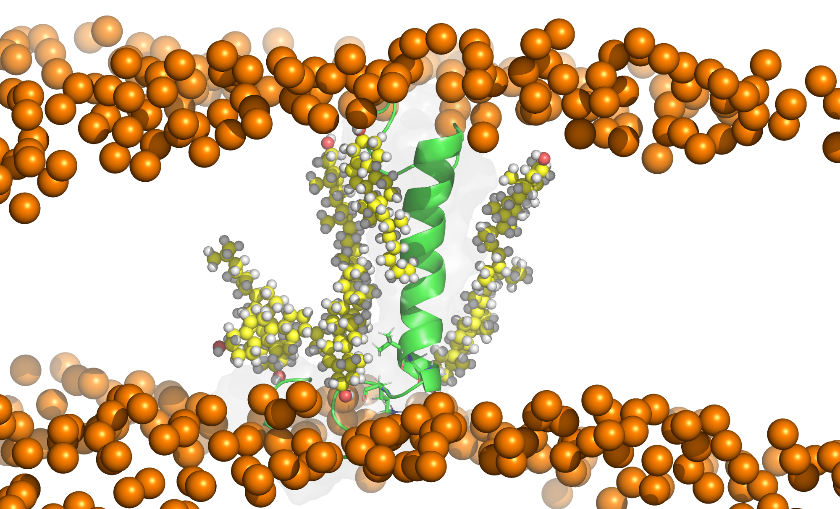


*Figure S8: A representative image of coarse-grained simulation remapped into all-atom resolution, showing cholesterol (yellow balls) accumulation near transmembrane helix (green cartoon). Orange balls represent phospholipid phosphate atoms. Other lipid tails and waters are omitted for clarity.*
